# An Ecological Definition and Objective Threshold for Differentiating Small Fragments

**DOI:** 10.1002/ece3.73054

**Published:** 2026-02-03

**Authors:** David C. Deane, Cang Hui, Melodie McGeoch

**Affiliations:** ^1^ Research Centre for Future Landscapes and Department of Ecological, Plant and Animal Sciences La Trobe University Melbourne Victoria Australia; ^2^ Centre for Invasion Biology, Department of Mathematical Sciences Stellenbosch University Stellenbosch South Africa; ^3^ Biodiversity Informatics Unit African Institute for Mathematical Sciences Cape Town South Africa; ^4^ Securing Antarctica's Environmental Future School of Biological Sciences, Monash University Clayton Australia

**Keywords:** biodiversity, fragmentation, mean‐species incidences, occupancy, patch size, range size, range‐rarity richness, small fragments, species representation

## Abstract

In an increasingly fragmented natural world, understanding how different ecological phenomena vary with patch size has many motivations. Examples include the assembly of biodiversity, ecosystem service provision and the suitability of fragments for habitat specialist species. A common approach to such questions divides fragments into small and large size classes for separate analysis. However, lack of an objective definition and means to differentiate ‘small’ from ‘large’ patches limits our ability to compare findings across studies, arguably impeding progress toward any unified views. Because larger and smaller fragments tend, on average, to respectively over‐represent narrow‐ and wide‐range species, an ‘area for unbiased species representation’ (A_USR_) can be defined at some intermediate fragment size predicted to contain species at incidence frequencies approximating that of the overall landscape. A central tendency for A_USR_ has previously been estimated for patchy habitats (islands, habitat islands and fragments), providing a benchmark to compare this threshold of small fragment size *between* studies. However, if A_USR_ can be readily determined *within* individual study systems, it would also provide an objective threshold to separate small and large fragments under the A_USR_ definition. Here we assess this potential for 138 published datasets from various fragmented landscapes using an index comparing species incidence frequencies in each fragment with that of the overall landscape. Regressing this index on fragment area yielded an estimate for A_USR_ in over 90% of cases, suggesting broad applicability as an objective way to separate fragments into two size classes. Regression slopes provide further information on the relative representation of narrow‐ vs. wide‐range species, with ~80% being numerically consistent with the overall negative trend. Requiring only the same data as the island species‐area relationship, A_USR_ can provide useful insights on the relative importance of narrow‐ vs. wide‐ranging species for studies of patch‐size dependence in ecological phenomena.

## Graphical abstract

Many ecological phenomena vary with fragment size. To quantify the impacts of small fragments on ecological pattern and process, one must differentiate them, but there is no agreed way to achieve this. We present an objective definition and method to identify a threshold size for small fragments that requires minimal data and can be readily calculated using the R code provided.

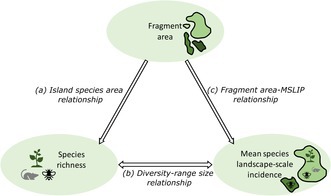

## 1 Introduction

In an ever‐more fragmented natural world, understanding the role of patch size on ecological phenomena is an increasingly urgent task (Jacobson et al. 2019). The most well researched of these phenomena is the maintenance of biodiversity in landscapes (i.e., fragmentation per se; Fahrig 2017), which is of enduring controversy (e.g., Fahrig et al. 2019; Fletcher et al. 2018). However, research on patch size dependence is also increasingly concerned with other questions, including ecosystem service delivery (e.g., Valdés et al. 2019), the distribution of generalist vs. specialist species (e.g., Volenec and Dobson 2020) and food web structures (e.g., Martinson and Fagan 2014). A common approach to questions of this nature classifies habitat patches as ‘small’ or ‘large’ and compares the magnitude of the phenomenon of interest between the two size classes. Typically, such classifications are based on the empirical patch size distribution, which limits our ability to generalise from one study system to the next even if an objective criterion (e.g., median fragment size) is used to differentiate ‘small’ and ‘large’ fragments. The lack of an ecological definition of a ‘small’ habitat fragment for a multi‐species community is thus an inherent challenge in revealing the importance of patch‐size effects in ecology. Herein we propose such a definition based on comparison of species composition in each fragment with that of the overall landscape. We then illustrate how this provides an ecologically defined benchmark to delineate small from large habitat fragments that requires minimal data.

**FIGURE 1:**
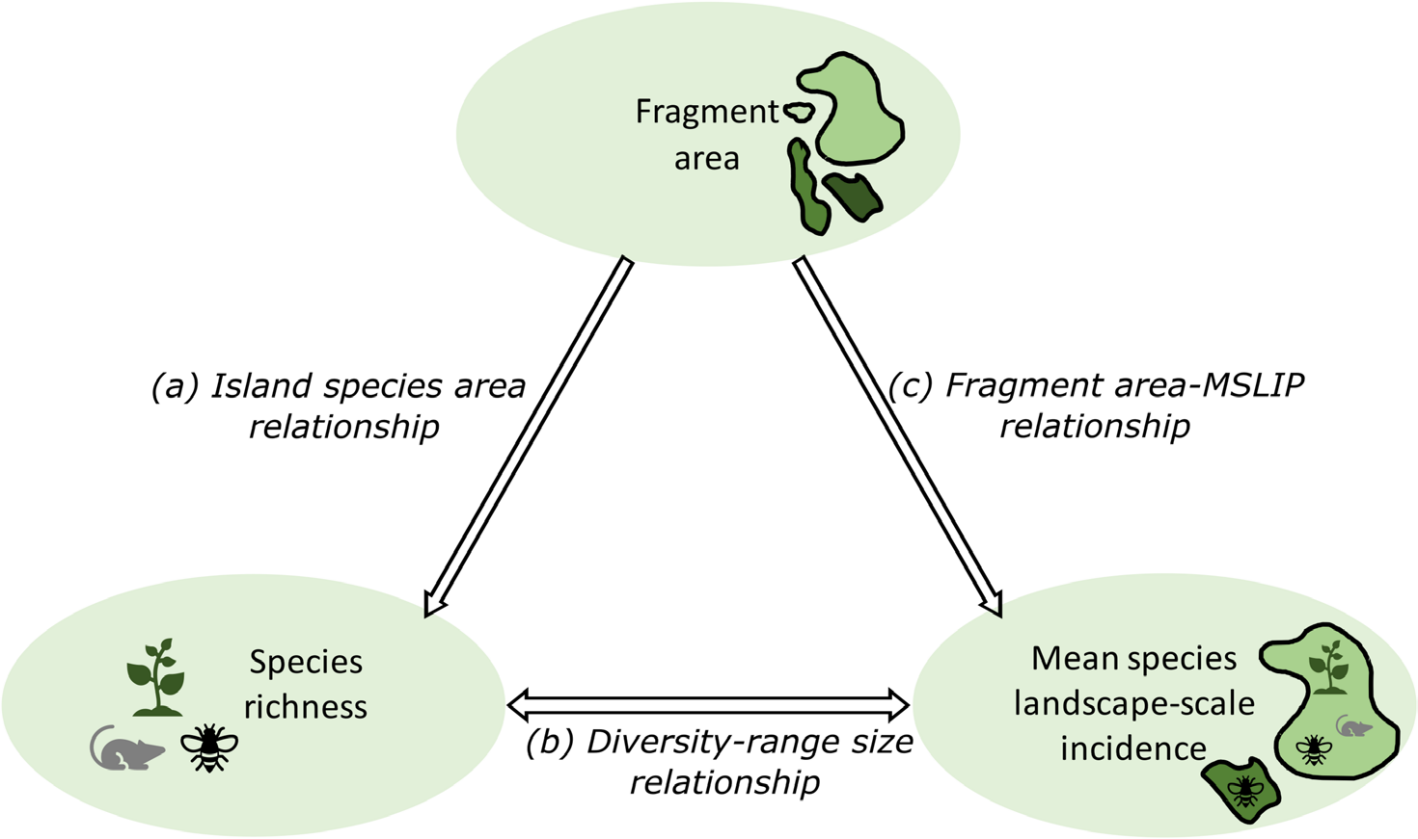
Interdependent relationships linking fragment area, the number of species they contain, and the average rarity of those species in the landscape. Adapted from Deane et al. (2024).

**FIGURE 2:**
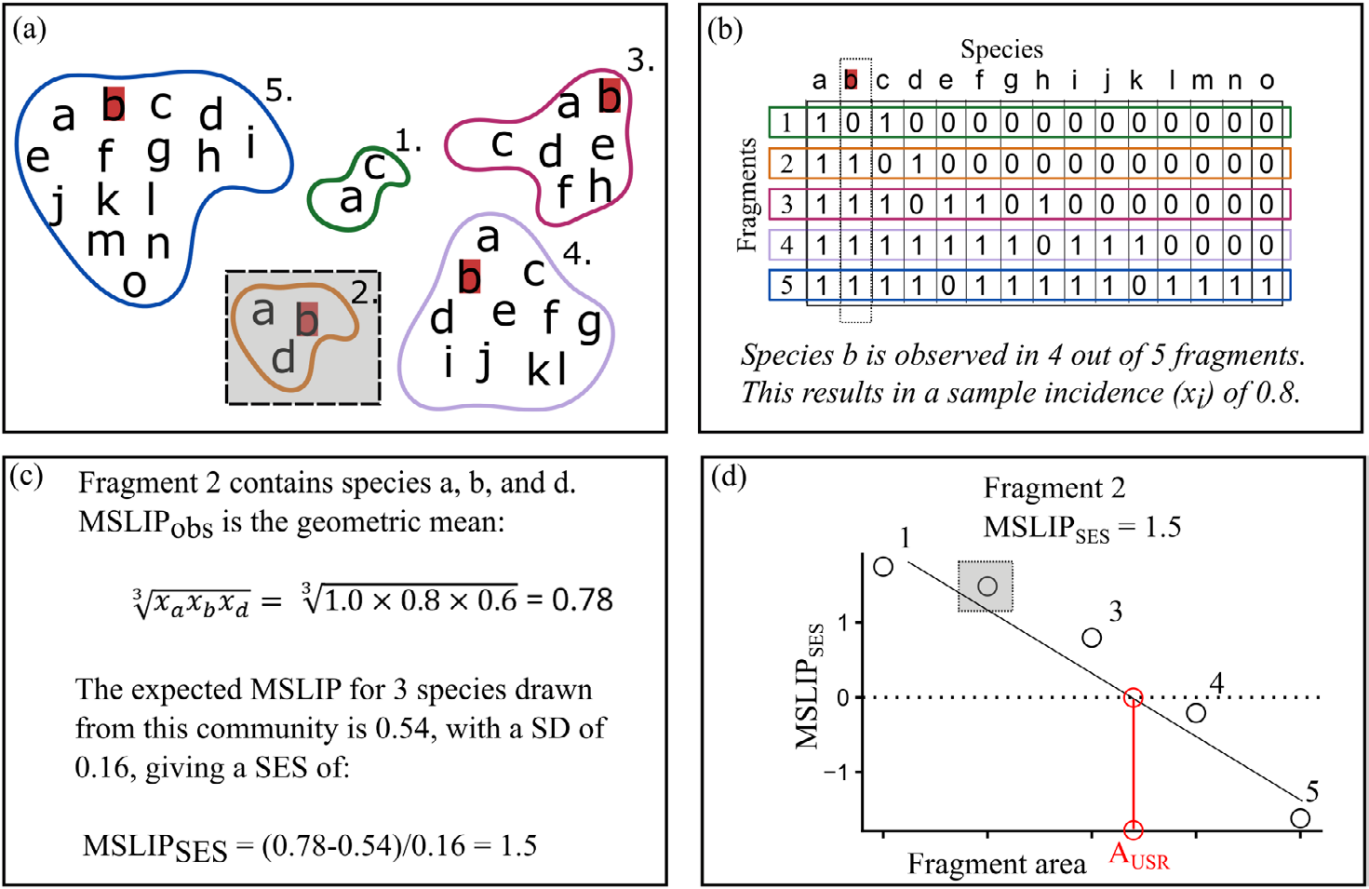
Calculation of the Mean Species Landscape‐scale Incidence per Patch (MSLIP) metric. Adapted from Deane et al. (2024).

Our thinking was initially inspired by reflection on the systematic conservation planning approach, which—for species representation—typically seeks to identify a minimum number of sites to protect the maximum number of species. Sites (or fragments) are prioritised according to both the number of species they contain (i.e., species richness) and their relative rarity (Margules and Pressey 2000). Sites with more rare species attract a higher weighting because of their elevated extinction risk (Myers et al. 2000). Yet, prioritising habitat to protect rarer species can have disproportionately high negative impacts on aggregate habitat availability for more common species (Neeson et al. 2018), despite their ecological importance (Baker et al. 2019) and emerging reports of their disproportionately rapid decline (Inger et al. 2015; Jansen et al. 2020). A complementary conservation goal might then seek to select patches to ensure a somewhat proportional representation of all species in the landscape, from the rarest to the most ubiquitous. However, this would ideally require data on the relative abundance of all species across that landscape, which are rarely available. One can, however, achieve something like this using only empirical presence‐absence data, by comparing the balance between wide‐ and narrow‐range species within each fragment against that of the overall landscape (Figures 1 and 2; Deane et al. 2024). As we show, such an approach has some useful applications, including an objective basis to delineate small and large habitat fragments.

The area of a discrete habitat fragment is associated with certain a priori ecological expectations (Box 1). For example, although it is debated (Fahrig 2020; but see Deane 2022), smaller fragments are often associated more with generalist than specialist species (Matthews et al. 2014; Smith et al. 2018). More generally, compared with the incidence frequency of all species across the landscape, larger fragments on average contain more narrow‐range species than expected, smaller fragments more widespread species (Deane et al. 2024). An important corollary of this relationship is that at some intermediate patch size, fragments are expected to contain a statistically similar composition of narrow‐ and wide‐range species as that of the overall landscape (Box 2). It is this intermediate‐sized ‘area for unbiased species representation’ (A_USR_) (Deane et al. 2024) that we propose as a possible objective threshold to separate habitat fragments within a single study system into two size classes.

BOX 1 **Interdependence of Fragment Area, Species Richness, and Species Rarity.**

The area of a fragment influences the expectation for the species it will contain via three interacting relationships (Figure 1): (a) the *island species area relationship* (ISAR) predicts larger fragments will contain a greater number of species; (b) the *diversity‐range size relationship* predicts that the more species a fragment contains, the smaller the average global range size of those species will be (Guo et al. 2022); and (c) the *fragment area‐mean species landscape‐scale incidence relationship (area‐MSLIP)* (Deane et al. 2024).

The latter relationship was derived from recognising that larger fragments contain more species, thus, if the diversity‐range size relationship holds, larger fragments should also contain species with a smaller average range size. Here, range size is estimated at the landscape‐scale, where one uses species incidence within that landscape (the number of fragments in which they occur) to quantify their rarity in the landscape. This led to a prediction (Deane et al. 2024) that smaller patches will contain, on average, species of higher mean landscape‐scale incidence (i.e., more widespread species in that landscape), for which a new fragment (or patch) scale index was developed called the mean species landscape‐scale incidence per patch (MSLIP), which is based on the geometric mean of the incidences (see Box 2 and Deane et al. 2024 for details).

The final piece of the puzzle is to recognise that the island‐species area relationship (ISAR) does not perfectly predict species richness: fragments of equal area can fall above or below the ISAR curve, respectively reflecting more or fewer species than expected for a fragment of that size in that landscape. This acts to mediate the expected MSLIP away from that expected based purely on fragment area. Thus, to fully understand the role of fragment size in selecting species from the landscape, one needs to consider all three relationships.

Whereas Deane et al. (2024) confirmed these interdependent relationships are consistent across true islands, habitat islands and fragments, they only tested for a general area‐MSLIP relationship across hundreds of datasets. No attempt was made in that study to explore the consistency in the pattern for individual fragmented landscapes. This would, of course, be needed to support its use for differentiating fragments into two size classes based on empirical species composition and is the primary aim of this study.

BOX 2 **Calculation of the Mean Species Landscape‐Scale Incidence Per Patch (MSLIP) Metric.**

The fragment—or patch—scale metric, *Mean Species Landscape‐scale Incidence per Patch* (MSLIP) characterises the incidence of species found within a fragment relative to that one would expect based on the incidence of all species across the landscape (Deane et al. 2024). To illustrate calculation of the metric, first consider a landscape comprising 5 fragments of varying size (shown by colour coded polygons in Figure 2a).

A common way to present such information is using a fragment‐by‐species (represented as letters within the polygons) presence‐absence matrix (Figure 2b). The row sums then correspond to the species richness of each fragment, while the column sums represent the incidence of each species across all fragments in the landscape. Species ‘b’, for example, is observed in 4 out of 5 fragments, yielding an incidence of 0.8 (Figure 2c).

Taking the geometric mean of the incidences of all species in each fragment yields a value for the observed MSLIP for each fragment. For focal fragment 2 (shown in shading in Figure 2a) this results in a value of 0.78 (Figure 2c). However, to place this value in the context of the broader landscape, one wishes to know how likely a value of this magnitude would arise, given the number of species observed in the focal fragment and the distribution of all species across all fragments. To achieve this, a resampling procedure is used to generate a reference distribution from which a standardised effect size for the observed MSLIP can be calculated as: ${\text{MSLIP}}_{\mathrm{SES}}=\left({\text{MSLIP}}_{\mathrm{obs}}-\bar{{\text{MSLIP}}_{\mathrm{ref}}}\right)/{\mathrm{SD}}_{\mathrm{ref}}$, that is, the difference between the observed MSLIP, the mean of the reference distribution normalised by the standard deviation of the reference distribution. For fragment 2, the expected value from 1000 draws of 3 species incidences (sampled with replacement) yields a mean of 0.54 and standard deviation of 0.16, thus the MSLIP_SES_ = 1.5. Positive values for MSLIP_SES_ indicate the fragment contains more widespread species than expected, conditional on the number of species observed and the incidence of all species across the landscape.

The final step is to fit a regression model of MSLIP_SES_ on fragment area (Figure 2d; see Appendix S2 for worked calculation). Where the regression line intercepts the 0 value for the y‐axis, one obtains an estimate for the area for unbiased species representation (A_USR_; shown in red, Figure 2d). This is the fragment size where species are expected to be sampled at a rate consistent with the incidences of all species across the landscape and is the value proposed as a threshold differentiating small from large fragments.

By synthesising evidence across multiple studies, A_USR_ has been estimated for patchy habitats (islands, habitat islands, fragments) (Deane et al. 2024), offering a benchmark to compare ecosystem specific thresholds of small fragments *between* study systems. However, for A_USR_ to be usefully applied as an objective threshold of small fragments *within* individual study systems, the ubiquity of the relationship between species representation and fragment area must be established. The aims of this study are to test the relationship between mean landscape‐scale incidence (a patch‐scale prevalence measure of narrow vs. wide‐range species representation; see Box 2) and fragment size among independent study systems and to explore the sources of any variation. Here we focus on patches that represent fragments of formerly continuous habitat rather than other discrete patch types because of the increasing interest in the explicit incorporation of small fragments for biodiversity conservation and ecosystem service provision (e.g., Arroyo‐Rodriguez et al. 2020). Additionally, we take the opportunity to provide an estimate of A_USR_ at the level of common fragmented habitat types (e.g., forest, grassland, etc.). Results suggest A_USR_ has potential as an objective threshold to delineate small and large fragments either at the level of individual study systems or as a benchmark measure of central tendency for comparison across different fragmented habitat types. Moreover, the slope of the proposed regression equation for quantifying A_USR_ provides a further basis for comparison, offering insights into the nature of the distribution of wide‐ and narrow‐range species across a fragmented landscape.

## 2 Methods

### 2.1 Background

Our study systems (or landscapes, these terms are used interchangeably) are habitat fragments of varying size, each with either one or more pooled samples (e.g., quadrats) or species lists. Represented in a fragment‐by‐species presence‐absence data matrix, columns record the incidence of species within each fragment (Box 2). We use the term ‘incidence’ to differentiate these data from grid‐based studies (which use ‘occupancy’) and from global range size estimates. Low incidence species are narrow‐range at the landscape scale (found in few fragments); high incidence species are wide‐range (found in many fragments).

The response variable was the *mean species landscape‐scale incidences per patch* (MSLIP), derived and validated in Deane et al. (2024). This metric compares the geometric mean of the landscape‐scale incidences of the residing species in each fragment with an expectation based on the incidences of all species in that landscape. This is converted to a standardised effect size (SES) to account for differences in species number in each fragment and the frequency distribution of incidences for all species in the study system. Positive (negative) MSLIP_SES_ values indicate the fragment contained more wide‐ (narrow‐) range species than expected. A zero value indicates that the fragment is representative of the landscape, so the fragment area where the MSLIP_SES_–area regression crosses the *x*‐axis (i.e., where MSLIP = 0) predicts the fragment size that contains an unbiased sample of species (in terms of incidences) from the landscape (i.e., the ‘area for unbiased species representation’, A_USR_). Box 2 illustrates the calculation, and the R code used to calculate MSLIP is provided in the Supporting Information. Note that to estimate MSLIP one needs to account for deviations from the island species‐area relationship (ISAR) as this also influences species representation within a patch (Deane et al. 2024).

### 2.2 Sources of Data

To model A_USR_ in fragmented landscapes, we combined two source databases. The first of these was a subset (78/202) of the database collated from the literature on discrete metacommunities (islands, habitat islands and fragments) (described in Deane 2022; Deane et al. 2024). To increase sample size, we added 60 datasets from the FragSAD database (Chase et al. 2019), available from the Dryad data repository (https://doi.org/10.5061/dryad.595718c, August 2019 version, accessed 8 December 2020). All datasets included metadata on broad *taxonomic group* (birds, invertebrates, non‐avian vertebrates and plants), *fragment type* (‘forest’, ‘grassland’, or ‘island’, respectively forest or woodland fragments within a terrestrial matrix, grass/shrub‐dominated fragments within a terrestrial matrix, and forest habitat fragments isolated by water due to reservoir creation), and *survey type* (‘standardised’ where all fragments had the same sampling approach, irrespective of fragment area; and ‘effort controlled’ where field sampling was adjusted more or less proportionally according to fragment size, increasing for larger fragments). To capture these variations in the level of confidence that data represent a near‐complete census for each fragment, a four‐level categorical indicator of *data confidence* was assigned, ranging from Level 1 for datasets based on field verified atlas data to Level 4 for standardised sampling without varying effort control for fragment size (Table S1; Deane 2022). The effects of standardised vs. effort adjusted sampling on outcomes were further explored in the individual‐level comparisons (see “Variation among study systems”). In total, 138 metacommunity datasets were available for modelling individual landscapes and for meta‐regression across habitats.

### 2.3 Modelling

#### 2.3.1 Variation Among Study Systems

**FIGURE 3:**
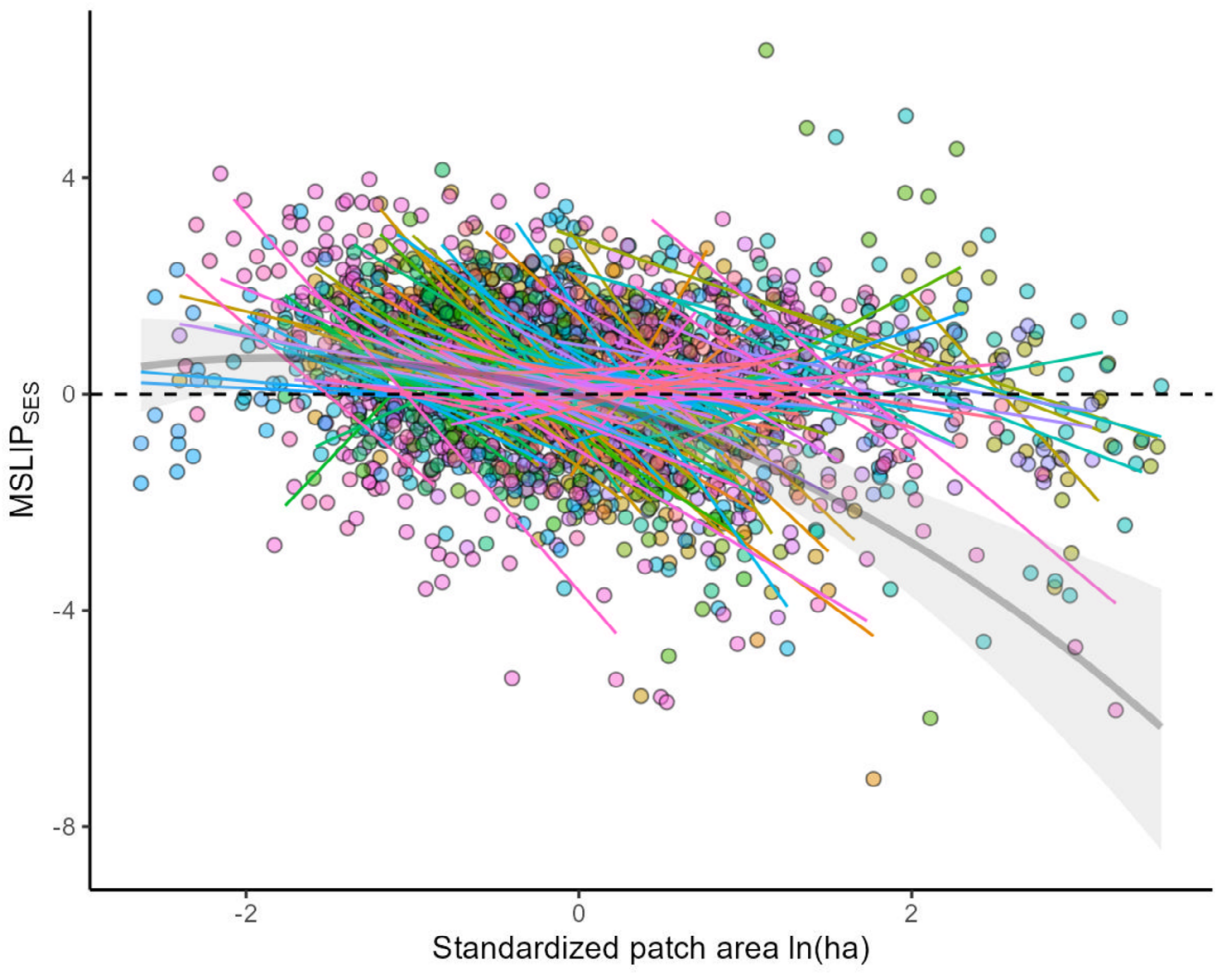
MSLIP‐fragment size relationship for individual study systems. Points show raw data and lines show best fit prediction from linear regressions (both are colour coded by fragment type). For reference, the median predictions of the overall curvilinear model (see Figure 2a) are overlain as partially transparent black solid line, with 50% credible intervals indicated by shading. An x‐axis value of zero corresponds to a fragment area of 8.7 ha.

To test the generality of the negative MSLIP_SES_‐area relationship for individual study systems (see Figure 3), and to explore possible sources of variation in this (see Table 2), each metacommunity dataset was first modelled separately, using linear regression with normally distributed errors. To control for the expected negative slope between MSLIP_SES_ and relative species richness (Deane et al. 2024), we included a covariate calculated as the residual deviation (*res*) of the observed species richness (${\mathrm{SR}}_{\mathrm{Obs}}$) in each fragment from that expected. For effort controlled data, this was predicted from an empirical power law island species‐area relationship (ISAR) $\left({\mathrm{SR}}_{\text{pred}}=c\cdot {\text{area}}^{z}\right)$ for that metacommunity (Deane et al. 2024), $\mathrm{res}=\left({\mathrm{SR}}_{\mathrm{Obs}}-{\mathrm{SR}}_{\text{pred}}\right)/{\mathrm{SR}}_{\text{pred}}$. For datasets that employed standardised sampling (where sampling effort was identical in all fragments), it would be unrealistic to expect the observed species richness to vary according to fragment size, precluding the use of the ISAR to compare relative richness. Thus, for the standardised datasets, *SR*_pred_ was the mean richness across all fragments. The residual deviation was then calculated as the difference between the observed richness and the mean over all fragments. Regardless of the method used to calculate it, negative *res* values indicate a species‐poor fragment relative to the expected richness. Quadratic and interaction terms (as used in meta‐regression—see ‘Variation among fragmented habitat types’) were omitted to simplify interpretation and avoid overfitting and the expected MSLIP_SES_ for fragment i in each metacommunity dataset was modelled as:

$${\text{MSLIP}}_{i}=\beta_{0}+\beta_{1}\left(\ln.{\text{area}}_{i}\right)+\beta_{2}\left({res}_{i}\right)+\varepsilon_{i},\;\varepsilon_{i}\sim N\left(0,\sigma \right).$$

The mean of the posterior distribution for the area coefficient (*β*_1_) was then used to explore among‐study variation, where we extracted the area coefficient (mean and 95% credible intervals) from each linear regression. Metacommunities where the mean slope was negative (consistent with the general pattern) or positive were identified, with the mean area coefficient used for visualising the predicted relationship over the range of fragment sizes for that metacommunity.

Mean area coefficients were then used to explore variation among study systems, using generalised least squares regression implemented in R package nlme (Pinheiro, Bates, DebRoy, Sarkar, and Team 2019). We included categorical predictors for broad taxonomic group and fragment type along with a binary predictor indicating standardised vs. effort‐controlled sampling design. To capture variations in the correlation between richness and area (‘SR‐area’) we used the *z*‐value of the ISAR for effort‐controlled studies and the Pearson correlation between *res* and fragment area for standardised data. Variations in the shape of the occupancy frequency distribution (OFD) were modelled using the truncated power law form of the ranked species occupancy curve (Hui 2012; Jenkins 2011), $O_{R}=aR^{b}e^{-\mathrm{cR}}$, where *O*_*R*_ is the occupancy of species ranked *R*, and *a*, *b*, and *c* are model parameters. Bimodality, as inferred from a positive fitted value for parameter *b* (Hui 2012), was used as a binary predictor of OFD shape. To account for heteroscedastic variance, we used an exponential variance structure allowing ‘SR‐area’ to vary by taxonomic group. To infer support for each of the predictors we used a Type 3 analysis of variance (Fox and Weisberg 2019) and estimated regression coefficients and categorical levels were compared using least squares means calculated using R package emmeans (Lenth 2019).

#### 2.3.2 Variation Among Fragmented Habitat Types and Other Covariates

To estimate the effects of fragmented habitat types, taxonomic groups, databases, data confidence on MSLIP_SES_‐area relationships, we used meta‐regression, analysing all metacommunity datasets within a single model. Model structures followed Deane et al. (2024) after confirming suitability with the new database (Appendix S1, Figure A1.1). We used a Bayesian multilevel model implemented with Hamiltonian Monte Carlo (HMC) sampling using Stan (Carpenter et al. 2017), coded using R package brms (Burkner 2017). The model was parameterized using weakly regularising priors and sampled using 4 independent chains each with 2000 iterations (1000 warm‐up). Convergence was confirmed via inspection of the HMC chains, ensuring all potential scale reduction factor (R‐hat) values ≤ 1.01, and all bulk effective sample sizes > 600. Model performance was assessed using Bayesian *R* ^2^ (Gelman et al. 2019).

Model structure included a varying (random) intercept and varying (random) slopes for (log‐transformed) fragment area and residual species richness for each study system (i.e., metacommunity dataset, indexed by *j*). Level 1 (fragment‐level) predictors were fragment area (*ln.area*), residual species richness (*res*), their interaction, and a quadratic term for fragment area. Level 2 (metacommunity‐level) categorical predictors were fragment type (*pch =* ‘forest’, ‘grassland’, or ‘island’), and taxonomic group (*tax =* birds, invertebrates, non‐avian vertebrates, or plants), both having interactions with the average fragment area of a metacommunity. Noting differences in response between the two data sources (Figure A1.1, Appendix S1), database was also included as a categorical predictor (*FS_db* indicating FragSAD datasets), allowing each to have a different slope by including an interaction with the average fragment area of a metacommunity. Because the MSLIP_SES_ distribution exhibited left skew, we used a skew‐normal response distribution and identity link. Finally, to account for heteroscedasticity, the variance (sigma) parameter in the response distribution was modelled Residual heteroscedasticity was modelled explicitly by allowing the log‐variance of the skew‐normal response distribution to vary as a function of ordinal confidence level using polynomial contrasts, with an additional dataset‐level random effect to capture unexplained between‐study variation in residual dispersion, as a function of the level of data confidence (*conf*), qualitatively assigned to ordinal categories from L1 (high) to L4 (low) (Deane 2022; Supporting Information), which were encoded using orthogonal polynomial contrasts (conf_L, for linear trend; conf_Q for quadratic curvature; conf_C for cubic deviation).

Thus, for fragment *i* (= 1, …, *N*_*j*_) in study system *j* (= 1, …, 138) the final model structure has the following form (see Table 1 for further description):

$${\text{MSLIP}}_{\left[i\right]j}\sim \mathrm{SN}\left(\mu_{\left[i\right]j},\sigma_{j}^{2},\alpha \right)$$

**TABLE 1:** Notation used in meta‐regression model and description of terms.

Notation	Description
${\text{MSLIP}}_{\left[i\right]j}$	Observed MSLIP value for fragment *i* in metacommunity *j*, modelled using a skew‐normal (SN) distribution
$\mu_{\left[i\right]j}$	Level‐1 (fragment‐level) conditional mean of MSLIP for fragment *i* in metacommunity *j* (*j* = 1, …, 138)
$\beta_{\mathrm{xj}}^{\mu }$	Level‐1 coefficient for predictor *x* in the mean MSLIP model; may vary among metacommunities
$\varepsilon_{\left[i\right]j}$	Fragment‐level residual error term
$\gamma_{0x}$	Level‐2 (metacommunity‐level) coefficients for the global mean MSLIP response. This includes the overall intercept $\left(\gamma_{00}\right)$ and effects of categorical predictors $\left(\gamma_{01}-\gamma_{07}\right)$ *Note:* Level‐2 categorical predictors are denoted using summation notation indexed by *t*. For example, $\sum_{t=1}^{2}\gamma_{0t}\left({\mathrm{pch}}_{t,\mathrm{jk}},\mathrm{ref}={\text{Frag}}_{\text{lake}}\right)=\gamma_{01}\text{forest}+\gamma_{02}\text{grassland}$, where *forest* and *grassland* are contrasted against the reference level *lake* (${\text{Frag}}_{\text{lake}}$)
$\gamma_{1x}$	Level‐2 coefficients describing how groups from metacommunity‐level categorical predictors modify the slope of MSLIP‐area (*ln_area*) relationship in response
$\gamma_{2x}$	Level‐2 coefficients describing how groups from metacommunity‐level categorical predictors modify the slope of MSLIP against residual deviation from expected species richness (*res*) in response
$u_{\mathrm{xj}}$	Coefficient for variation among datasets, where *x* = 0, 1 and 2 denoting intercept, slope for fragment area and slope for residual deviation respectively
$\tau_{\mathrm{xy}}$	Variance (x=y) or covariance (x≠y) between the level‐2 intercept (*x*, *y* = 0) and slopes (1 = *ln_area*, 2 = *res*)
$\beta_{0j}^{\sigma }$	Coefficients for data confidence level. Coded as an ordinal factor (L, Q and C refer to the linear, quadratic and cubic levels of the polynomial contrasts)
$u_{j}^{\sigma }$	Random variation in model variance among datasets

Level‐1 (fragment‐level) structure:

$$\mu_{\left[i\right]j}=\beta_{0j}^{\mu }+\beta_{1j}^{\mu }\left(\ln.{\text{area}}_{\left[i\right]j}\right)+\beta_{2j}^{\mu }\left({\mathrm{res}}_{\left[i\right]j}\right)+\beta_{3}^{\mu }\left(\ln.{{\text{area}}^{2}}_{\left[i\right]j}\right)+\beta_{4}^{\mu }\left(\ln.{\text{area}}_{\left[i\right]j}\times {\mathrm{res}}_{\left[i\right]j}\right)+\varepsilon_{\left[i\right]j}.$$

Level‐2 (metacommunity‐level) structure:

$$\beta_{0j}^{\mu }=\gamma_{00}+\sum_{t=1}^{2}\gamma_{0t}\left({\mathrm{pch}}_{t,j},\mathrm{ref}={\text{Frag}}_{\text{lake}}\right)+\sum_{t=3}^{5}\gamma_{0t}\left({\mathrm{tax}}_{t,j},\mathrm{ref}=\text{birds}\right)+\gamma_{06}\left(\mathrm{FS}\_{\mathrm{db}}_{j}\right)+\gamma_{07}\left(\mathrm{FS}\_{\mathrm{db}}_{j}\times {\bar{\ln.{\text{area}}^{2}}}_{j}\right)+u_{0j},$$

$$\beta_{1j}^{\mu }=\gamma_{10}+\sum_{t=1}^{2}\gamma_{1t}\left({\mathrm{pch}}_{t,j},\mathrm{ref}={\text{Frag}}_{\text{lake}}\right)+\sum_{t=3}^{5}\gamma_{1t}\left({\mathrm{tax}}_{t,j},\mathrm{ref}=\text{birds}\right)+\gamma_{16}\left(\mathrm{FS}\_{\mathrm{db}}_{j}\right)+u_{1j},$$

$$\beta_{2j}^{\mu }=\gamma_{20}+u_{2j},$$

where the errors are assumed to be correlated and to follow a multivariate normal (MVN) distribution,

$$\left[\begin{array}{c} u_{0j}\\ u_{1j}\\ u_{2j} \end{array}\right]\sim \mathrm{MVN}\left[\left(\begin{array}{c} 0\\ 0\\ 0 \end{array}\right),\left(\begin{array}{ccc} \tau_{00} & \tau_{10} & \tau_{20}\\ \tau_{10} & \tau_{11} & \tau_{21}\\ \tau_{20} & \tau_{21} & \tau_{22} \end{array}\right)\right].$$

Variance model structure:

$$\log\sigma_{j}^{2}=\beta_{0j}^{\sigma }+\beta_{1j}^{\sigma }\left(\text{conf}\_L\right)+\beta_{1j}^{\sigma }\left(\text{conf}\_Q\right)+\beta_{1j}^{\sigma }\left(\text{conf}\_C\right)+u_{j}^{\sigma },$$

**FIGURE 4:**
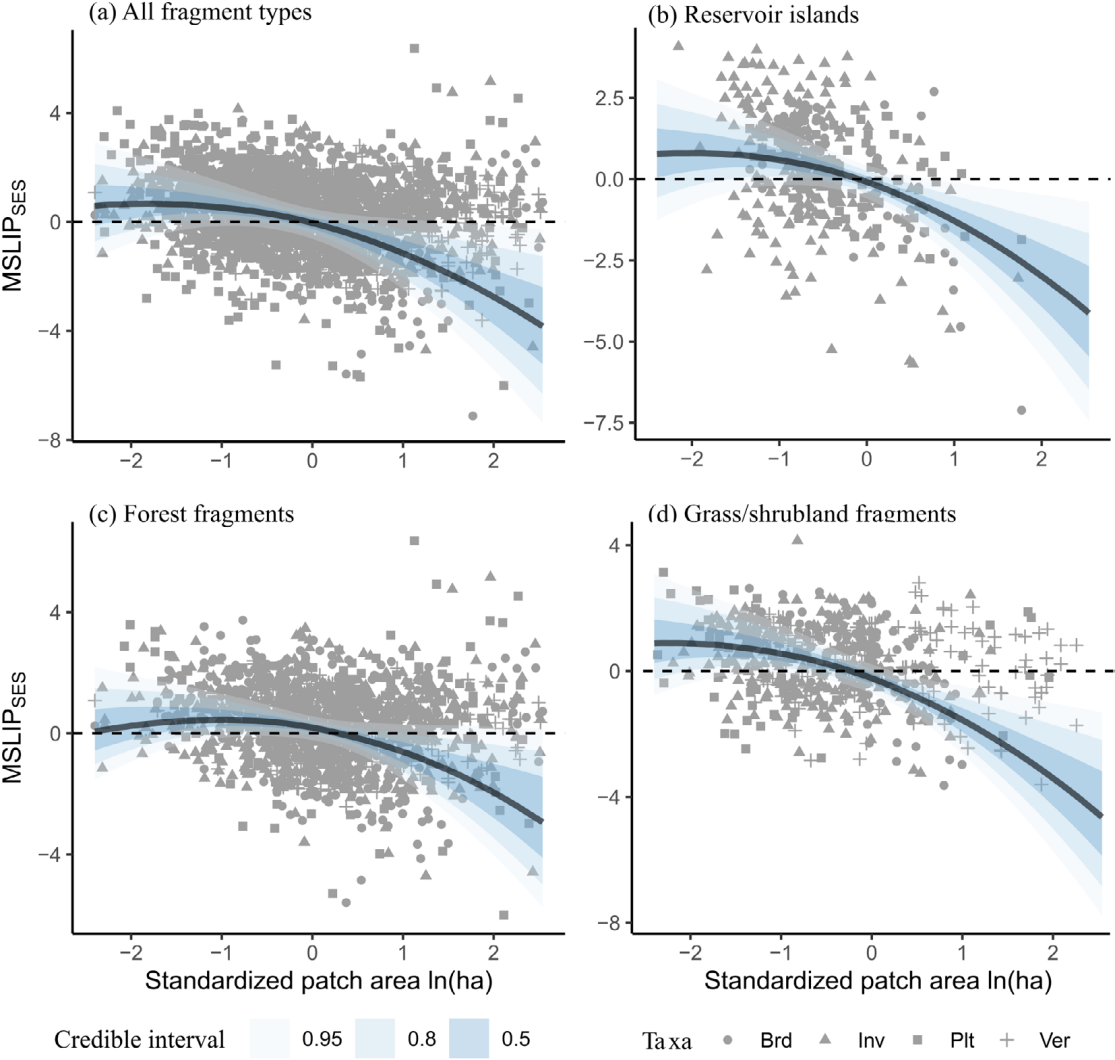
Variation in mean species incidences per fragment (MSLIP) as a function of fragment area for different fragment types. Modelled standardised effect sizes (MSLIP_SES_) for (a) pooled response over all fragment types, (b) reservoir islands, typically forested hilltops fragmented by flooding (c) forest fragments, and (d) grassland fragments. Curves show posterior predictions (median and credible intervals) from the model. Model predictions are averaged over all taxonomic groups and assume that fragment richness follows the species area relationship (i.e., *res* = 0). See Table 2 for the range of area values and estimated area for unbiased species representation for each fragment type. An *x*‐axis value of zero corresponds to a fragment area of 8.7 ha.

Posterior predictive distributions (PPD) from the meta‐regression model were used to estimate the area for unbiased species representation among different fragment types (see Figure 3) and among different taxonomic groups for forest fragments (see Figure 4). We included uncertainty in model parameters but not group‐ or population‐level variance in PPD. All PPD used to estimate A_USR_ were averaged over source database, data confidence and assumed all fragments had expected richness (i.e., *res* = 0). For A_USR_ among different fragment types, PPD were also averaged over taxonomic groups, while variation in A_USR_ among taxonomic groups was only estimated for forest fragments (which contained the most balanced replication across taxonomic group levels). To estimate A_USR_ uncertainty, we used leave‐one‐out cross validation, refitting the model with one dataset omitted and re‐estimating A_USR_. Note A_USR_ estimates here differ from those in Deane et al. (2024), as areas for many of the discrete island and habitat‐island types used (e.g., oceanic archipelagos), were much larger than fragments (e.g., the mean fragment size of 58 ha, compares with 8.7 ha here). This affects model predictions due to shrinkage to the global mean area. We anticipated A_USR_ estimated only for fragmented habitats would be smaller, but also more robust for fragmented habitats, than the Deane et al. (2024) estimates.

## 3 Results

At the level of individual study systems (Figure 3), regression explained on average around half of the variation in MSLIP_SES_ (Bayesian *R* ^2^ = 0.55 [0.21, 0.88]; mean [95% CI]). The MSLIP_SES_‐area regression line intersected the zero line (allowing an A_USR_ estimate) for 125 of the 138 metacommunity datasets (90.6%). There was no apparent feature that distinguished the 13 studies where the regression did not cross the zero line (e.g., in terms of taxa and matrix dimension), although their universally small absolute slope values (|median slope| = 0.3 vs. 1.5; Kruskal‐Wallis test, *χ* ^2^ = 25.0, df = 1, *p* < 0.001) indicate relatively weak association between species representation and fragment area. The slope of the MSLIP_SES_‐area relationship was negative in 109 of the 138 study systems (79%), with 95% CI excluding zero for 56 of these. The remaining 29 study systems had a positive slope (one excluding zero).

Around 20% of total variation in slope was explained by predictors (Table A1.1, Appendix S1), with higher positive correlation between species richness and fragment area (predominantly due to smaller ISAR exponents), and standardised survey effort in all fragments, both decreasing the slope (*p* < 0.006; Table 2, Table A1.2). There was weaker evidence of differences among taxonomic groups (*p* = 0.011), driven by more negative coefficients (indicating greater over‐representation of wide‐range species in small fragments) in birds than non‐avian vertebrates (*p* = 0.008; Table A1.2). There was no evidence fragment type or bimodality in the occupancy frequency distribution influenced the slope in the MSLIP_SES_‐area relationship (both *p* > 0.15; Table 2).

**TABLE 2:** Relationships between predictors and the slope of the MSLIP_SES_‐fragment area relationship.

Predictor	Df	*χ* ^2^	Pr (> F)
Intercept	1	0.86	0.354
SR‐area correlation	1	12.2	< 0.001
Bimodality	1	2.09	0.148
Fragment type	2	0.51	0.777
Taxonomic group	3	11.1	0.011
Standardised sampling design	1	7.69	0.006

*Note:* SR‐area correlation = the slope of the power law island species area relationship (for datasets that adjusted effort according to fragment area) or the Pearson correlation between deviations from mean fragment richness and fragment area (for datasets with equal sampling effort in all fragments); Bimodality = 1, if occupancy frequency distribution was bimodal (inferred from a positive b parameter in the power‐exponential function fit to occupancy data); Fragment type = 3 level categorical variable (levels: islands, forests, or grass/shrublands); Taxonomic group = 4 level categorical variable (levels: birds, invertebrates, plants, vertebrates); Standardised sampling design = binary predictor, where 1 indicates equal sampling effort per fragment regardless of size.

**FIGURE 5:**
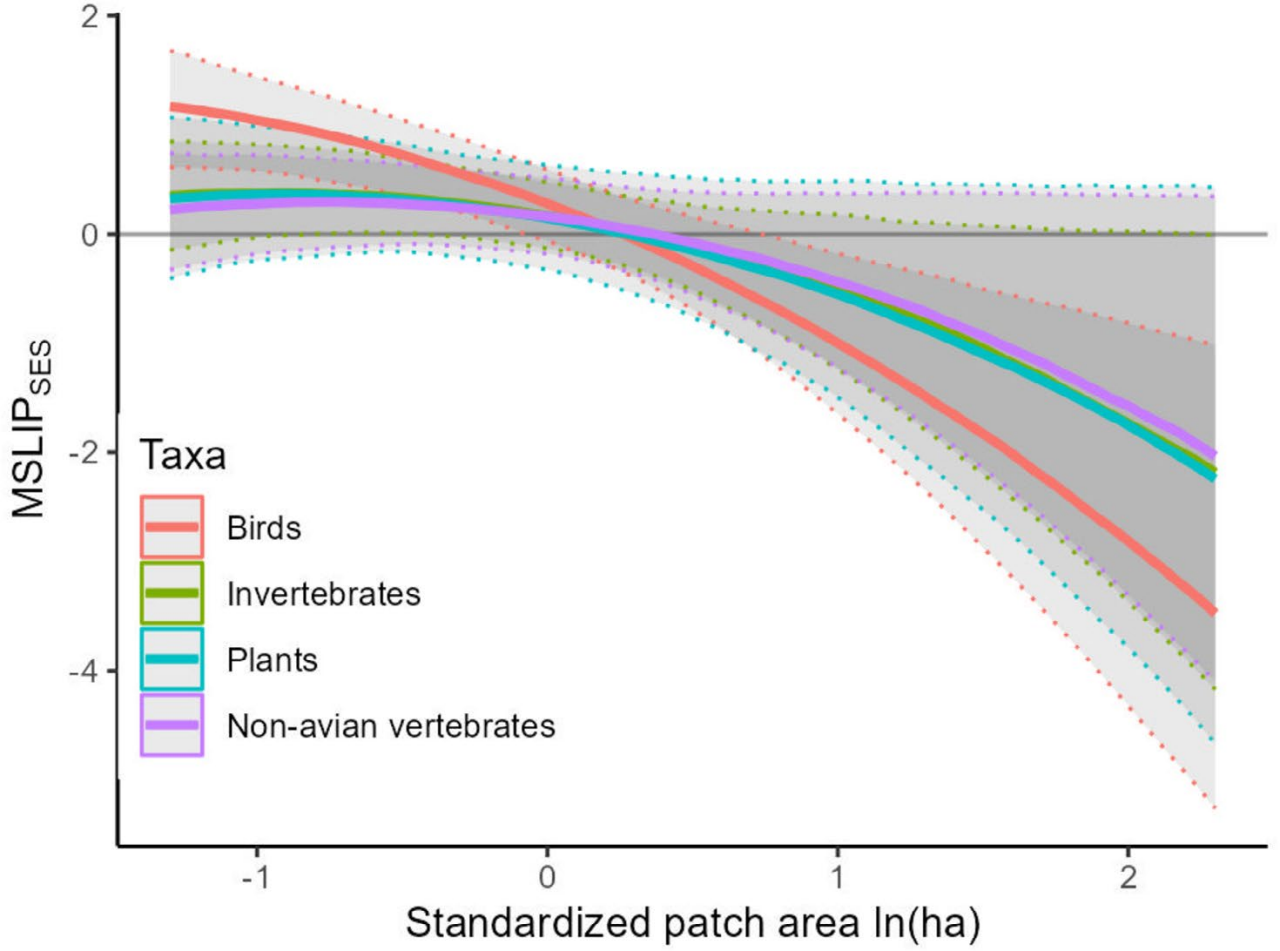
Taxonomic group differences in the MSLIP‐fragment size relationship for forest fragments. Line colours indicate taxonomic group, shading indicates 95% credible interval, with boundaries indicated by a dotted line. Model predictions assume that fragment richness follows the species area relationship (i.e., *res* = 0). An *x*‐axis value of zero corresponds to a fragment area of 8.7 ha.

At the level of fragmented habitat type, the MSLIP_SES_‐area relationship was a qualitatively identical, negative curvilinear function of fragment area (Figure 4, Table 3; Figure A1.2) and regression explained nearly half of the variation (Bayesian *R* ^2^ = 0.46 [0.43, 0.49]; mean [95% CI]). A model with the quadratic term omitted had less than 0.1% support relative to the full model (leave‐one‐out cross validation). A positive median MSLIP_SES_ value was evident for even the smallest modelled fragment landscapes (~0.1 ha), although reservoir islands (Figure 4b) and grass/shrubland fragments (Figure 4d) had higher MSLIP_SES_ than forest/woodland fragments (Figure 4c). Averaged over all taxonomic groups, the estimated area (in hectares) for unbiased species representation (A_USR_) varied between 4.6 [4.1, 5.8] for grass/shrubland and 19.9 [18.8, 21.4] for forest fragments (median and 95% sampling interval; Table 2). MSLIP_SES_‐area relationships were also qualitatively similar for taxonomic groups (Figure 5), although for forest fragments birds had the highest representation of wide‐range species at small fragment sizes, despite having the smallest A_USR_ (16.6 ha; non‐avian vertebrates were largest at 22.2 ha; Figure 5). Deviations from the expected fragment species richness according to the ISAR (*res*) altered the balance between narrow‐ and wide‐range species observed at constant fragment size, such that relatively speciose fragments contained, on average, more narrow‐range species regardless of their area (Figure A1.3).

**TABLE 3:** Fragmented habitat types (no. datasets) and their fragment areas with the median area associated with unbiased species representation (A_USR_, i.e., the fragment area where mean occupancy of species in that fragment approximates the mean occupancy of all species in the metacommunity).

Fragment type	Range in fragment area (ha) median [90% CI]	A_USR_ (ha) median [95% CI]
Reservoir islands (7)	1.1 [0.2, 48.0]	6.6 [6.1, 7.1]
Forest (105)	14.1 [0.3, 4585.5]	19.3 [18.0, 20.7]
Grassland (26)	3.5 [0.1, 400.0]	4.6 [4.1, 5.3]
All fragments (138)	8.8 [0.1, 4585.5]	7.7 [7.3, 8.4]

## 4 Discussion

We propose the lack of any clear definition of what constitutes a small fragment as a key impediment to the study of patch‐size dependence in ecological phenomena. Regression of the MSLIP_SES_ on fragment area (Figure 2, Appendix S2) offers a potential solution, providing an objective means to differentiate ‘large’ from ‘small’ fragments based on the area for unbiased species representation (A_USR_). We found A_USR_ can be readily calculated in over 90% of study systems, suggesting it has widespread potential for application in differentiating small from large fragments. Importantly, this threshold has an ecologically grounded definition: the area at which fragments are predicted to contain species at incidence frequencies approximating that of the overall landscape. In addition to the A_USR_ area threshold, though, the direction and magnitude of the slope of the MSLIP_SES_‐area regression (Figure 2, Appendix S2) provide a convenient metric of species representation for comparison among landscapes. Of particular interest are the characteristics of study systems where the slope is positive as this implies rare species are over‐represented in small patches (~20% of cases). This situation could arise where a high proportion of species are confined to a single patch, an expectation for highly fragmented landscapes comprising many small fragments (Deane et al. 2022), or a combination of highly specialised taxa and fragments that show strong abiotic differentiation (Deane et al. 2023). Studies with slopes near zero (~9% of cases) are also of interest as these imply no patch‐size dependence on species representation, although this could also possibly be due to a relatively homogeneous fragment size distribution. The MSLIP_SES_‐area relationship (and A_USR_) requires only presence‐absence species lists (e.g., from timed meander surveys); thus, data requirements are low. Notably, these same data allow the island‐species area relationship to be estimated, and both offer complementary information for interpreting area‐based ecological phenomena.

For individual fragments, deviations from the ISAR are important to account for because the mean incidence of species is co‐determined by both the area and the relative species richness of a fragment (Box 1); fragments with richness exceeding the ISAR prediction are likely to contain more narrow‐range species than expected for their size. This phenomenon, which follows from the negative diversity‐range size relationship (Guo et al. 2022), has implications for optimisation algorithms based on species representation (e.g., range‐rarity richness relationships; Guerin and Lowe 2015). Specifically, range‐rarity richness corrected by local species richness will potentially fail to identify optimal sites (Martin‐Fores et al. 2021), because higher range‐rarity (numerator) is partially offset by higher species richness (denominator). Thus, at the landscape level, not only are shallower ISAR slopes (e.g., *z* < ~0.3) associated with greater species density among groups of smaller, relative to larger, fragments for equal total area (Deane 2022; Liu et al. 2022), they also increase the probability of observing narrow‐range species in smaller fragments (indicated by the negative regression coefficient; Figure A1.2, Appendix S1).

### 4.1 A_USR_ as a Landscape‐Scale Fragment‐Size Benchmark

Rather than representing a target for reserve network design or universal conservation policy threshold, we view A_USR_ as a benchmark with both landscape‐ and habitat‐scale applications. At the landscape‐scale, it is common practice for studies to split fragments into ‘small’ and ‘large’, often based on their relative value for generalist and specialist species (e.g., Murphy et al. 2022; Rosati et al. 2010; Smith et al. 2018; Yan et al. 2023), or to compare their contributions to ecosystem services (Decocq et al. 2016; Valdés et al. 2019). For any such study, estimating A_USR_ for that landscape provides an objective point of comparison for area‐based classification of fragments.

Similarly, if landscape conservation goals foster explicit protection of large and small fragments (Arroyo‐Rodriguez et al. 2020; Rösch et al. 2015), the A_USR_ for that landscape might be used to delineate small patches. Alternatively, A_USR_ for a landscape could be viewed as a benchmark of intermediate‐size fragments, which have experimental (Loke et al. 2019), theoretical (Ovaskainen 2002), and empirical (Lawrence et al. 2018) support as being optimal for biodiversity conservation. An interesting question that arises in this context is whether groups of fragments close to the predicted A_USR_ would result in an incidence frequency distribution like the landscape, but we lack the data to verify this. The key general point to appreciate is that any focus on preserving (or removing) *either* large *or* small fragments will likely result in changes to the incidence frequency distribution of species in that landscape. Whether this is desirable will be context dependent; calculating A_USR_ for that landscape will help establish this context.

Finally, it is worth considering the MSLIP‐area relationship in the context of the small island effect (SIE), where islands (habitat islands, patches) below a given size do not follow an island species area relationship (Lomolino and Weiser 2001). Various explanations have been offered to account for this phenomenon, including disturbance, habitat diversity, and extinction rates (summarised in Chisholm et al. 2016). If over‐representation of widespread species partially contributes to the SIE, then the area for unbiased species representation might offer an independent estimate of the break in slope for the ISAR. Another possibility arises when the quadratic term is included in the MSLIP‐area regression, where the curve can intersect the zero line at very small fragment size. Although typically this only occurs for habitat islands (Deane et al. 2024), in cases where it is observed, the two crossings could be interpreted as offering threshold areas for both small and large fragments. On the other hand, because MSLIP is a patch‐scale index of species composition, empty fragments will not have a defined value, which might limit the use of the MSLIP‐area curve in SIE research (Wang et al. 2016).

#### 4.1.1 Applications for Habitat‐Scale A_USR_ Estimates

Ecologists tend to be justifiably cautious of universal habitat thresholds (Huggett 2005; van der Hoek et al. 2015), and we recognise that in many fragmented landscapes all remaining native habitat is likely to be beneficial. For example, small, isolated fragments are known to provide valuable stepping stone habitat (Terborgh 1974), and are increasingly recognised for ecosystem service provision (Arroyo‐Rodriguez et al. 2020; Valdés et al. 2019). We also recognise that any threshold is unlikely to apply in every landscape (Tulloch et al. 2016). Here, the 1638 forest/woodland fragments (most < 600 ha) yielded an A_USR_ estimate of ~20 ha. While this is near the global *mean* forest fragment estimate of 13–17 ha, the *median* is similar to 0.09 ha (Taubert et al. 2018). In many fragmented forest or woodland landscapes, 20 ha might already be considered large. Similarly, for the 592 grass/shrubland fragments (most < 97 ha) included, estimated A_USR_ was around 5 ha, which might be considered large in many landscapes (Rösch et al. 2015).

On the other hand, if one aims to understand why variation in patch‐size dependence arises across landscapes, an indicative area range to define small fragments in different fragmented habitat types could be useful. We suggest fragmented habitat type estimates of A_USR_ (i.e., Table 3) are best viewed as a null‐hypothesis, for comparison across studies. For example, 10 ha has been suggested as a threshold fragment size for maintenance of vascular plant diversity in oak forests near Rome (Rosati et al. 2010) and to support specialist birds in old growth forest mosaics in northern Sweden (Edenius and Sjöberg 1997). Against these values the forest A_USR_ estimate herein (~20 ha; Table 2) appears conservative, but this will not always be the case. Comparison of landscape and habitat‐scale estimates of A_USR_ could yield new insights. In grass/shrubland studies, 5 ha was recommended to preserve shrubland bird diversity (Shake et al. 2012) and 3–6 ha for area‐sensitive grassland birds (Walk and Warner 1999), while in fragments < 5 ha, specialist grassland plants are under‐represented in Mongolia (Yan et al. 2023). These values are consistent with A_USR_ for grassland as estimated here, suggesting that general guidance for delineating small and large patches might be reasonable, although further validation of A_USR_ for this purpose is clearly warranted. Another potential application is to compare the value of the MSLIP‐area slope and/or A_USR_ for the same set of fragments in a landscape at two points in time. Although we are not able to test this, one might expect the effects to vary according to the shape of the MSLIP‐area relationship. Depending on the direction of change, this could provide a line of evidence for relaxation (e.g., a steepening slope, decreasing A_USR_) or successful restoration of smaller fragments (e.g., shallower slope, larger A_USR_). Whether applied at habitat or landscape scales, for any study aiming to compare fragments based on their size, A_USR_ offers an objective benchmark to differentiate large and small fragments that is not data demanding and has a sound ecological basis.

## Author Contributions

**David C. Deane:** conceptualization (lead), data curation (lead), formal analysis (lead), investigation (lead), methodology (equal), writing – original draft (lead). **Cang Hui:** conceptualization (supporting), formal analysis (supporting), investigation (supporting), methodology (supporting), writing – original draft (supporting). **Melodie McGeoch:** conceptualization (supporting), formal analysis (supporting), funding acquisition (lead), investigation (supporting), methodology (supporting), project administration (lead), supervision (lead), writing – original draft (supporting).

## Conflicts of Interest

The authors declare no conflicts of interest.

## Supporting information


**Data S1:** ece373054‐sup‐0001‐Appendices.docx.

**Data S2:** ece373054‐sup‐0002‐Supinfo.docx.

## Data Availability

Data and code to replicate this analysis are archived in a Dryad repository (DOI: https://doi.org/10.5061/dryad.0rxwdbs89).
