# Supplementary material for: An Ecological Definition and Objective Threshold for Differentiating Small Fragments
Source: Ecol Evol. 2026 Feb 3;16(2):e73054. doi: 10.1002/ece3.73054 (PMC12865510; doi:10.1002/ece3.73054)
Supplement: Supplementary file 1 — Data S1: ece373054‐sup‐0001‐Appendices.docx. [file ECE3-16-e73054-s001.docx]

# Appendix S1 Supporting results


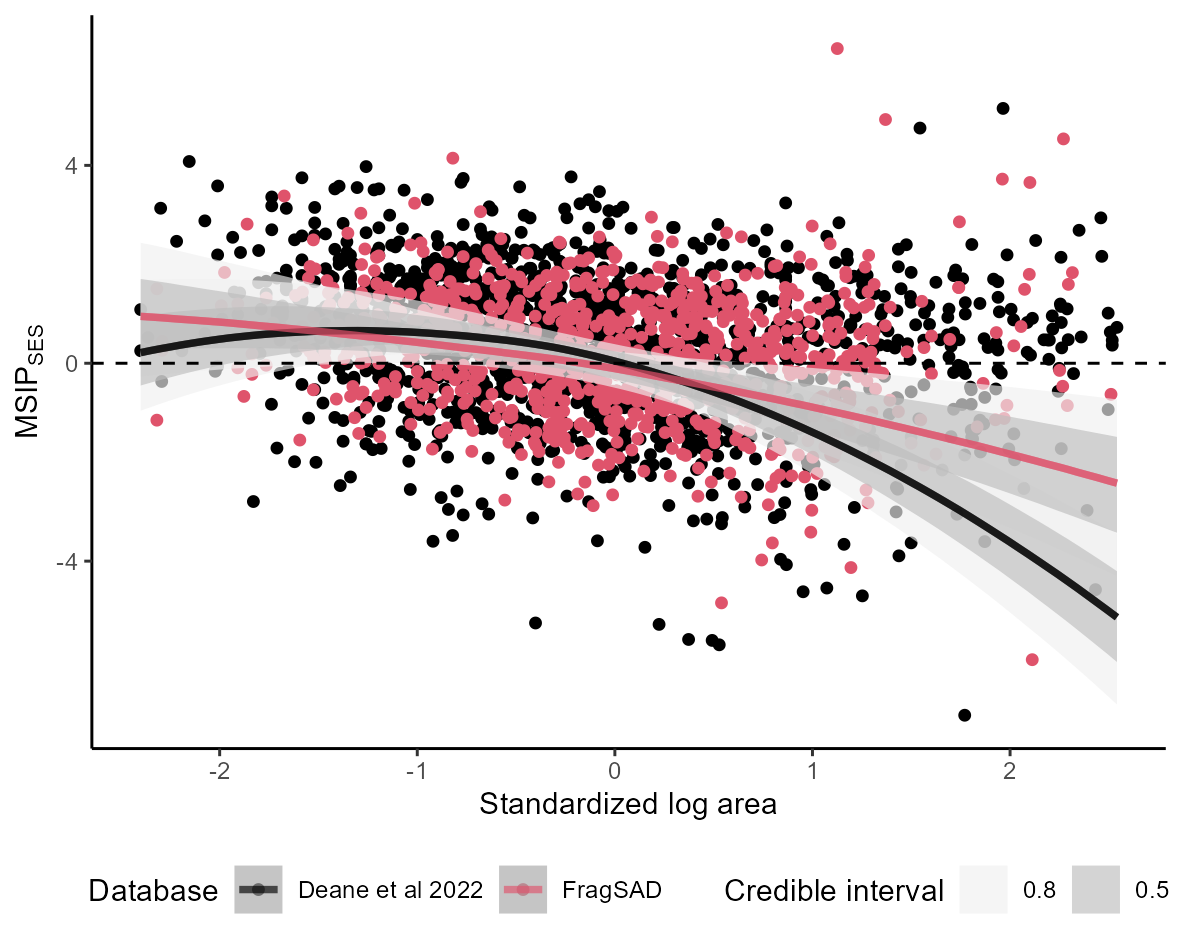


1. MSLIP-fragment size relationship modelled separately for the Deane (2022) and FragSAD databases.


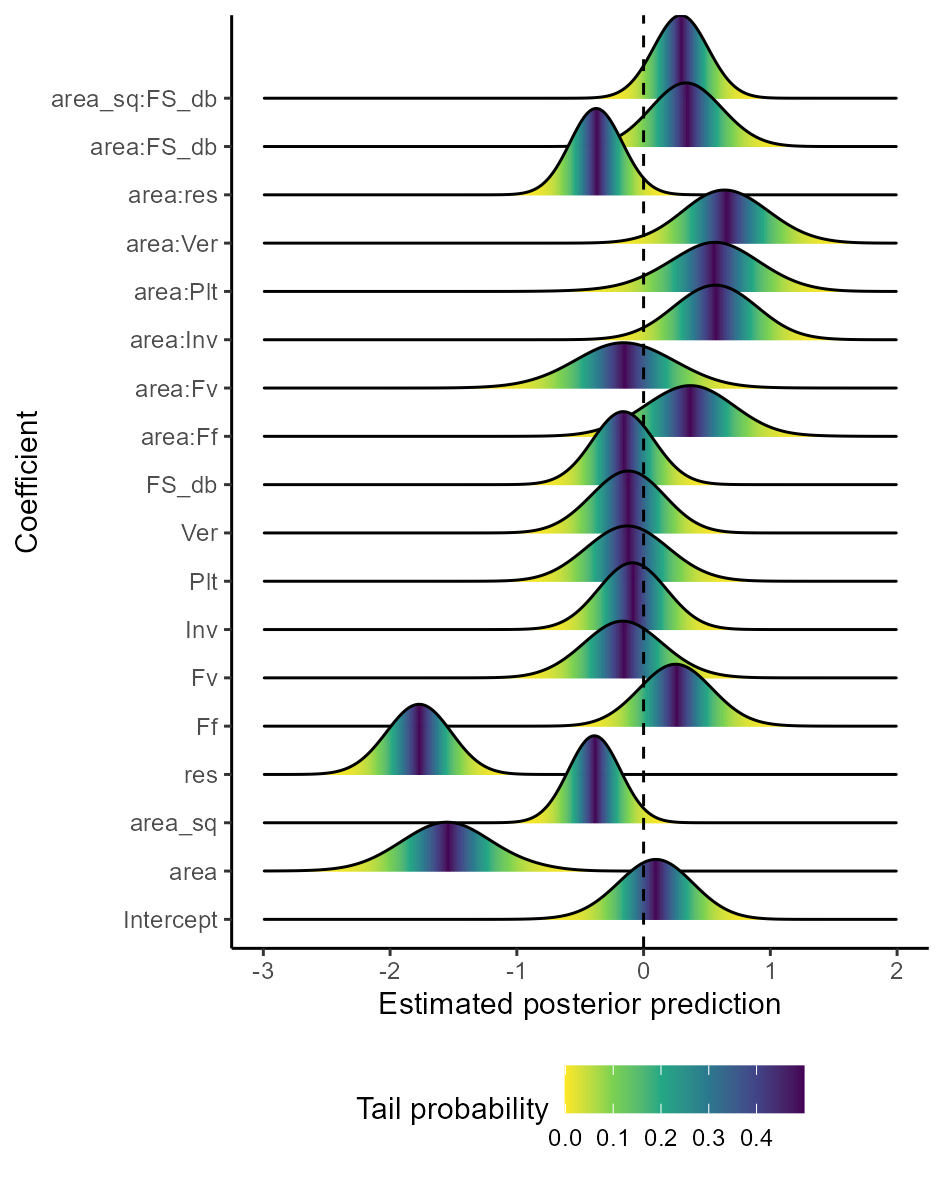


1. Posterior prediction for model coefficients, combined databases (*n* = 138). Coefficient abbreviations: *area* = natural log transformed area in hectares, *area_sq* = quadratic in area; *res* = residual deviation of fragment species richness relative to expectation for that metacommunity; Ff = forest fragments; Fv = other fragments (grass, shrubland); Inv = invertebrates, Plt = plants, Ver = non-avian vertebrates; FS_db = binary indicator that the metacommunity was from the FragSAD database. Colons indicate interaction terms. See main text for model equation.


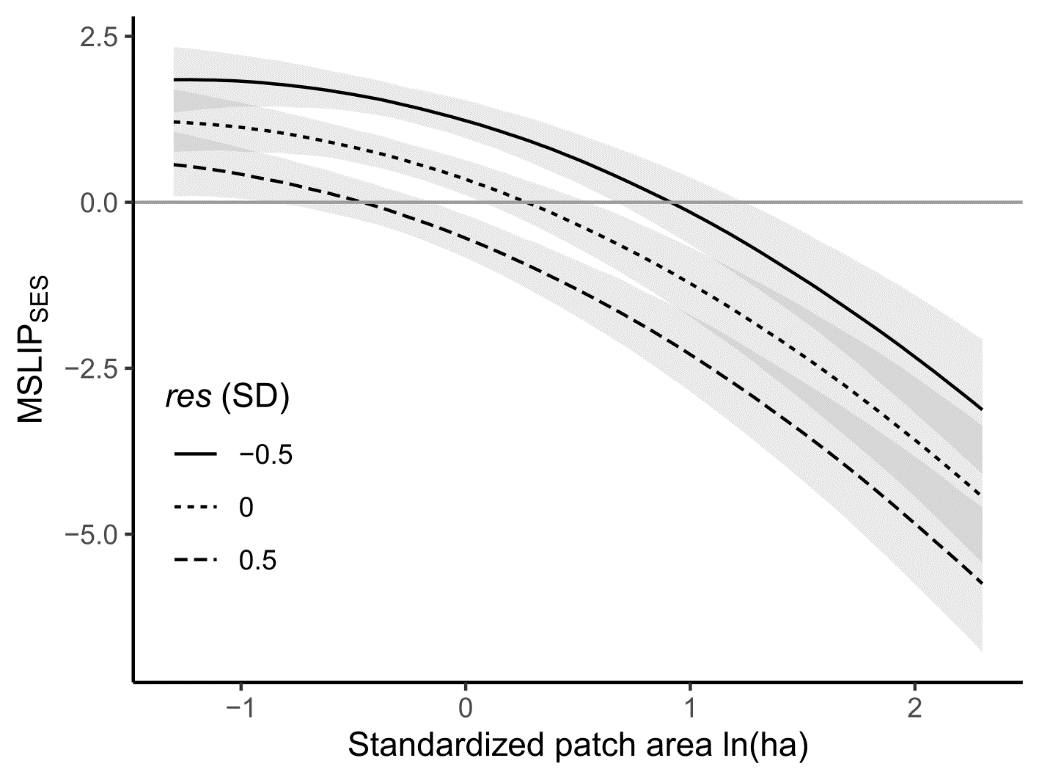


1. Effect of relative species richness on the fragment size-range size relationship for forest birds. Each line indicates the expected MSLIP value for fragments of equal area that either follow the island species area relationship (*res* = 0) or fall either half a standard deviation below (-0.5) or above (0.5) expected species richness. The area for unbiased species representation (i.e., A_USR_ where the modelled relationship crosses the zero *x*-axis) correspond to fragments of areas 2.4, 18.4 and 105 ha for *res* = 0.5 SD, 0, -0.5 respectively.

Table A1.1 Results of ordinary least squares regression for the origin of variation in the slope of the relationship between MSLIP_SES_ and fragment area for individual study systems

| *Predictors* | *Estimate* | *95% CI* | *P-value* |
| --- | --- | --- | --- |
| (Intercept) | -0.59 | -1.16 – -0.02 | 0.044 |
| SR-area correlation | -0.18 | -0.33 – -0.04 | **0.015** |
| Bimodality | 0.17 | -0.11 – 0.46 | 0.233 |
| Fragment type [Ff] | -0.19 | -0.75 – 0.36 | 0.492 |
| Fragment type [Fv] | -0.05 | -0.65 – 0.56 | 0.882 |
| Taxonomic group [Inv] | 0.40 | 0.01 – 0.80 | **0.045** |
| Taxonomic group [Plt] | 0.41 | -0.07 – 0.88 | 0.094 |
| Taxonomic group [Ver] | 0.77 | 0.38 – 1.15 | **<0.001** |
| Standardized sampling design | 0.94 | 0.60 – 1.27 | <0.001 |
| Observations | 138 | | |
| Pseudo R^2^ | 0.185 | | |

Table A1.2 Post hoc comparison of factor level differences among taxonomic groups. (a) estimated marginal means with results averaged over the levels of bimodality, fragment type, and standardized sampling design. (b) pairwise contrasts in marginal means. Degrees-of-freedom method: Satterthwaite, confidence level used: 0.95

| (a) Marginal means | | | | | |
| --- | --- | --- | --- | --- | --- |
| *Level* | *mean* | *SE* | *df* | *lower.CL* | *upper.CL* |
| Brd | -0.228 | 0.177 | 118.3 | -0.579 | 0.123 |
| Inv | 0.124 | 0.146 | 96.8 | -0.165 | 0.414 |
| Plt | 0.315 | 0.230 | 114.2 | -0.141 | 0.771 |
| Ver | 0.416 | 0.129 | 64.9 | 0.159 | 0.673 |
| (b) Pairwise contrasts in marginal means | | | | | |
| *Contrast* | *estimate* | *SE* | *df* | *t.ratio* | *p.value* |
| Brd-Inv | -0.353 | 0.210 | 128 | -1.682 | 0.3377 |
| Brd-Plt | -0.543 | 0.271 | 126 | -2.006 | 0.1911 |
| Brd-Ver | -0.644 | 0.198 | 124 | -3.251 | 0.0080 |
| Inv-Plt | -0.190 | 0.254 | 120 | -0.749 | 0.8768 |
| Inv-Ver | -0.291 | 0.200 | 120 | -1.454 | 0.4688 |
| Plt-Ver | -0.101 | 0.260 | 123 | -0.388 | 0.9801 |

# Appendix S2 Calculating MSLIP and A_USR_

***Calculating mean species landscape-scale incidences per patch***

In the main text, we modelled the fragment-level metric called the mean species landscape-scale incidences per patch (MSLIP_SES_). This metric has a generally negative relationship with fragment area. We propose using this concept to objectively identify a fragment area that provides roughly equivalent *potential* habitat value (in terms of species occupancy probability) for all species in that landscape, from the most widespread to the most narrowly distributed. We call this the area for unbiased species representation (A_USR_).

While we use the metric to predict a threshold A_USR_ applicable for different fragmented habitat types (e.g., forest, grassland, etc), all fragmented landscapes follow different area distributions, which might differ from the size range applied to estimate A_USR_ for that habitat. We therefore suggest that the MSLIP_SES_ ~ area regression be done at the landscape scale. To facilitate this, we provide a simple R function to calculate MSLIP_SES_. The function can be applied to either presence-absence or abundance data (although this is simply converted to p/a), provided fragment areas are available. The data format for the function is a fragments-by-species data frame, with fragment area in the first column, and the remaining columns containing species presence-absence in each fragment.

Below we illustrate the data format using the Gibson et al (2013) dataset (Near-Complete Extinction of Native Small Mammal Fauna 25 Years After Forest Fragmentation, Science 2013 Vol 341:1508-1510), which the authors used to evaluate mammal extinctions on reservoir island forest fragments of different sizes. These data are included in the FragSAD database.

To load the data, copy the file “Gibson_2013_dat.RData” to the working directory where the code is to be run (or add the path to the file in which it is located).

load(file="Gibson_2013_dat.RData")
library(knitr)
kable(gibdat[1:5, 1:5])

|  | area | Callosciurus caniceps | Chiropodomys gliroides | Echinosorex gymnurus | Maxomys surifer |
| --- | --- | --- | --- | --- | --- |
| island16 | 0.3 | 0 | 0 | 0 | 0 |
| island2 | 0.4 | 0 | 0 | 0 | 0 |
| island28 | 4.7 | 0 | 0 | 0 | 0 |
| island3 | 1.4 | 0 | 14 | 0 | 0 |
| island33 | 1.7 | 0 | 0 | 0 | 0 |

These data are in the format expected by the following function, used to calculate MSLIP_SES_. The function has two arguments ‘dat’ (the data) and ‘reps’ the number of resampling iterations to calculate MSLIP_SES_. It returns a dataframe object, with fragment area, species richness, MSLIP_SES_ and the raw value of mean species landscape-scale incidences (MSLIP_Obs_).

mslip.fn <- function(dat, reps = 100) {
 # Function to calculate the mean species landscape incidences per patch as derived in Deane et al (2024) GEB

# Args:

# dat = fragment (patch, island) by species dataframe, but with area in the first column, presence absence (or abundance) of species in the remaining columns (see example below for data format)
# reps = number of resampling draws from occupancy frequency distribution used for standardized effect size calculations (typically at least a couple thousand, depending on size of dataset. NB default set for convenience)
# Returns:

# outdf = dataframe with four columns: patch area in the 1st, fragment richness in 2nd, observed MSLIP in 3rd, MSLIP_SES in the 4^th^
# R code

I <- order(dat[,1])
 area <- dat[,1][I]

 spp <- ifelse(dat[I,-c(1)] > 0, 1,0)
 sr <- apply(spp,1,sum)
 ofd <- apply(spp,2,sum)
 leno <- length(ofd)

 nsite <- nrow(dat)
 ofdp <- ofd/nsite

 # calculate mean and SD of observed data
 pr.ave <- exp(mean(log(ofdp)))
 pr.sd <- exp(sd(log(ofdp)))

 # placeholders
 aver <- sdr <- mslip.obs<- mslip.ses <- c()

 set.seed(108)
 for(j in 1:nrow(spp)){
 len <- sum(spp[j,]>0) # number of presences
 mslip.obs[j] <- obs <- exp(mean(log(ofdp[which(spp[j,]>0)])))
 sims <- c()
 for(p in 1:reps){
 sam <- sample(ofdp, len, prob = ofdp, replace = TRUE)
 sims[p] <- exp(mean(log(sam),na.rm=TRUE))
 }
 aver[j] <- ave.rep <- mean(sims, na.rm=TRUE)
 sdr[j] <- sd.rep <- sd(sims, na.rm=TRUE)
 mslip.ses[j] <- (obs - ave.rep)/sd.rep
 }
 outdf <- data.frame(area=area, SR = sr, mslip.ses = mslip.ses, mslip.obs = mslip.obs)
 return(outdf)
 }

After loading the function, calculating MSLIP_SES_ is simple.

y <- mslip.fn(dat = gibdat, reps=100); kable(head(y))

|  | area | SR | mslip.ses | mslip.obs |
| --- | --- | --- | --- | --- |
| island16 | 0.3 | 1 | 1.3936471 | 1.0000000 |
| island2 | 0.4 | 1 | 1.4373580 | 1.0000000 |
| island40 | 0.8 | 1 | 1.2457648 | 1.0000000 |
| island39 | 1.0 | 2 | -0.0433132 | 0.4330127 |
| island41 | 1.1 | 3 | 0.6180186 | 0.5151607 |
| island3 | 1.4 | 2 | 0.5368224 | 0.5590170 |


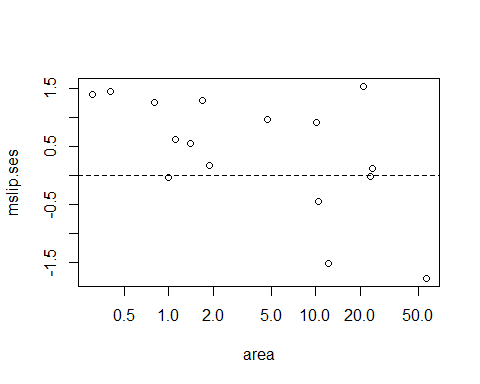


Because incidences are sensitive to the relative species richness of a patch, it is also recommended that differences from expected species richness are controlled for in the regression. Because the data here are species lists from fragments that differ in size, we use the island species area relationship (ISAR) to estimate an expectation and calculate the residual deviation of observed data from this value. First the ISAR (using the linearised power law ISAR).

lm.sar <- lm(log(SR) ~ log(area), data=y)
plot(log(y$SR) ~ log(y$area))
abline(lm.sar)


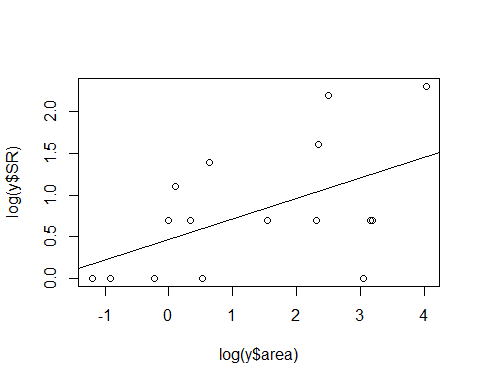


y$res <- (log(y$SR) - predict(lm.sar))/predict(lm.sar)

with(y, plot(mslip.ses ~ area, log="x")); abline(h=0,lty=2)

***Calculating the Area for unbiased species representation (A_USR_)***

The first step in calculating A_USR_ is to model the relationship between MSLIP_SES_, fragment area and residual deviation in species richness.

lm1 <- lm(mslip.ses ~ log(area) + res, data=y)
# plot(lm1) # typical check for model assumptions; here no serious patterns in residuals were apparent.

We then use the regression to predict the MSLIP_SES_ ~ fragment area curve and estimate the zero crossing. We can do this numerically by predicting MSLIP_SES_ for different areas and extract the value.

aseq <- seq(from = min(y$area), to = max(y$area), length.out=1000)
nd <- data.frame(area= aseq, res=0)
pred <- predict(lm1, newdata=nd)

# ... determine the zero MSLIP_ses crossing point (i.e., Ausr) from the prediction vector...
aseq[max(which(pred>0))]

## [1] 12.296

with(y, plot(mslip.ses ~ area, log="x")); abline(h=0,lty=2)
lines(aseq,pred)


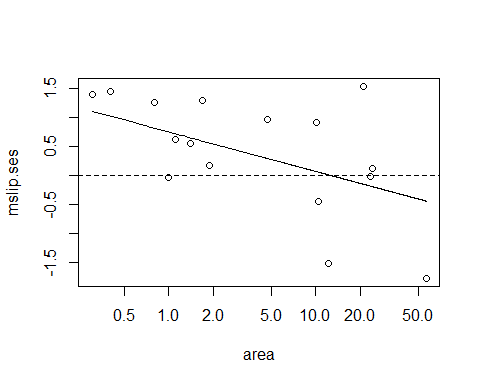


Or, in this simple case, we can also just use the regression coefficients to calculate the exact *x*-axis value for *y* = 0 (assuming *res* = 0) analytically.

fn1 = function(x) coef(lm1)[2]*x + coef(lm1)[1]
exp(uniroot(f = fn1, lower = min(y$area), upper = max(y$area))$root)

## [1] 12.30341

Here, the A_USR_ (corresponding to island fragment area) is around 12 ha. Qualitatively, this accords well with the authors’ conclusions for this system that time to extinction was greater for islands smaller than 10 ha. It is larger than our estimated A_USR_ for reservoir island fragments (6.6 ha), suggesting this value would be an underestimate for this specific set of island fragments.

Note that it is possible that the coefficient for area in the regression is positive - if this were to be found for empirical data, it suggests a relatively unusual system, where smaller patches over-represent narrowly distributed species. We found this in about 1 in 5 systems, although only for one of these did the slope differ conclusively from zero. The estimated A_USR_ is still a useful threshold to know (possibly more so) because it still provides an objective means to separate fragments such that the reasons for the fragment-size related patterns in distribution could be explored.

Additionally, if the coefficient for area (whether positive or negative) does not differ from zero, it is possible (albeit unusual) that the regression line will not cross the horizontal zero axis. In this case, experience suggests it is likely that A_USR_ is equal to the largest patch (assuming a negative slope), or alternatively, that there is no evidence of patch-size dependence in species representation for that taxon within that set of fragments (e.g., that fragments randomly sample species independently of their incidence).
