# Supplementary material for: An Ecological Definition and Objective Threshold for Differentiating Small Fragments
Source: Ecol Evol. 2026 Feb 3;16(2):e73054. doi: 10.1002/ece3.73054 (PMC12865510; doi:10.1002/ece3.73054)
Supplement: Supplementary file 2 — Data S2: ece373054‐sup‐0002‐Supinfo.docx. [file ECE3-16-e73054-s002.docx]

# Supporting information for

# An ecological definition of small fragments

## Metadata on source databases

**Table S1** Metadata on sources. Filename = unique identifier for database, summary = brief description of study system; taxo_group = broad taxonomic group used in modelling (Brd = birds, Ver = non-avian vertebrates, Inv = invertebrates; Plt = plant); metacom = broad metacommunity type (Af = reservoir island fragment; Ff = forest or woodland fragment, Fv = other vegetative fragment such as grass or shrubland); nfrag = number of fragments, nspp = total number of species; Country = location of study; minArea = smallest habitat patch area; maxArea = largest habitat patch area; areaUnit = unit of minArea, maxArea; Source = primary or grey literature from which data were obtained (see Data source publications); Conf = Integer score designating confidence in the likelihood data represent a full census for each fragment (1 = atlas or field confirmed atlas; 2 = multiple survey methods or collation of multiple field visits; 3 = single field survey effort adjusted 4. single survey no effort adjustment or validation or multiple surveys without effort adjustment to patch size [or precise methods unknown]); database = source of data used (dd = Deane et al (2024), fs = fragSAD).

| filename | summary | taxo | metacom | nfrag | nspp | Country | minArea | maxArea | areaUnit | Source | Conf | database |
| --- | --- | --- | --- | --- | --- | --- | --- | --- | --- | --- | --- | --- |
| Aguiar_2012 | Euglossine bees in forest fragments | Inv | Ff | 9 | 11 | Brazil | 2 | 18 | ha | Aguiar & Gaglianone 2012 | 4 | fs |
| Aizen_1994 | Feral Honey Bees in Argentine “Chaco Serrano” | Inv | Ff | 6 | 28 | Argentina | 0.5 | 3.8 | ha | Aizen & Feinsinger 1994 | 4 | fs |
| Almeida-Gomes_2014 | Lizards of Atlantic forest fragments | Ver | Ff | 9 | 7 | Brazil | 4.1 | 264 | ha | Almeida-Gomes & Rocha 2014 | 4 | fs |
| brdFFfPTt_Anciaes | Passerine birds in forest fragments | Brd | Ff | 5 | 59 | Brazil | 15 | 350 | ha | Anciaes & Marini 2000 | 2 | dd |
| invFFvPTt_Baldi | Orthopterans of shrub fragments in grassy matrix | Inv | Fv | 27 | 36 | Hungary | 0.18 | 40 | ha | Baldi & Kisbenedeck 1999 | 3 | dd |
| invFFfPTt_Baz | butterflies in fragments | Inv | Ff | 13 | 81 | Spain | 3.6 | 2115 | ha | Baz & Garcia-Boyero 1996 | 2 | dd |
| Bell_2006_a | frogs & lizards in forest fragments | Ver | Ff | 9 | 34 | Costa Rica | 1.43 | 6.88 | ha | Bell & Donnelly 2006 | 2 | fs |
| Bell_2006_b | frogs & lizards in forest fragments | Ver | Ff | 9 | 55 | Costa Rica | 1.43 | 6.88 | ha | Bell & Donnelly 2006 | 2 | fs |
| invFFfPTt_Bennedick | butterflies in fragments | Inv | Ff | 8 | 79 | Borneo | 120 | 122450 | ha | Benedick et al 2006 | 4 | dd |
| Berg_1997 | birds on forest fragments | Brd | Ff | 12 | 42 | Sweden | 3.6 | 13.2 | ha | Berg 1997 | 3 | fs |
| Bernard_2007 | bats on shrubland/steppe fragments | Ver | Fv | 10 | 33 | Brazil | 6 | 66 | ha | Bernard & Fenton 2007 | 2 | fs |
| brdFFfPTt_Blake2 | birds on isolated woodlots | Brd | Ff | 12 | 46 | US- Illinois | 1.8 | 600 | ha | Blake 1991 | 3 | dd |
| mamWFvUTt_Bolger | Mammals of chaparral fragmetns in an urban matrix | Ver | Fv | 25 | 9 | US-California | 0.41 | 84.1 | ha | Bolger et al 1997 | 4 | dd |
| brdFFfPTt_Bond | Breeding birds of upland forest fragments | Brd | Ff | 64 | 48 | US - Wisconsin | 6.1 | 202.3 | ha | Bond 1957 | 4 | dd |
| Bragagnolo_2007 | Harvestmen in Atlantic forest fragments | Inv | Ff | 8 | 17 | Brazil | 14 | 175 | ha | Bragagnolo et al 2007 | 4 | fs |
| Brosi_2008 | Bees of forest fragments | Inv | Ff | 19 | 114 | Costa Rica | 0.24 | 55.19 | ha | Brosi et al 2008 | 2 | fs |
| Brosi_2009 | Euglossine bees of forest fragments | Inv | Ff | 22 | 24 | Costa Rica | 0.24 | 296.49 | ha | Brosi 2009 | 4 | fs |
| hrpWFfPTt_Cabrera | Amphibians & reptiles in rainforest fragments | Ver | Ff | 6 | 35 | Mexico | 1.4 | 17.4 | ha | Cabrera-Guzman & Reynoso 2012 | 3 | dd |
| brdFFfPTt_Capizzi1 | birds in forest fragments 1996 | Brd | Ff | 17 | 31 | Italy | 0.1 | 40 | ha | Capizzi et al 2015 | 4 | dd |
| brdFFfPTt_Capizzi2 | birds in forest fragments 2006 | Brd | Ff | 17 | 33 | Italy | 0.1 | 40 | ha | Capizzi et al 2015 | 4 | dd |
| Carneiro_2016 | plants on forest fragments | Plt | Ff | 9 | 311 | Brazil | 20.91 | 87.18 | ha | Carneiro et al 2016 | 4 | fs |
| brdFFfPTt_Castellata | birds in forest frags | Brd | Ff | 17 | 167 | Singapore | 7 | 935 | ha | Castellatta et al 2005 | 2 | dd |
| Cayuela_2006 | Trees of forest fragments | Plt | Ff | 14 | 227 | Mexico | 71 | 5664 | ha | Cayuela et al 2006 | 3 | fs |
| mamWFfUTt_Charles | Small mammals in kerangas (forest frags) | Ver | Ff | 8 | 9 | Brunei | 0.01 | 22.8 | ha | Charles & Ang 2010 | 1 | dd |
| mamWFvUTt_Crooks | Small mammals of chaparral fragments in an urban matrix | Ver | Fv | 29 | 7 | US | 2 | 102 | ha | Crooks 2002 | 3 | dd |
| brdFFvUTt_Crooks | birds in chapparal fragments in an urban matrix | Brd | Fv | 27 | 7 | US - as per Soule and Bolger | 2 | 102 | ha | Crooks et al 2001 | 3 | dd |
| pltSFvUTt_Crowe | Weedy plants of urban vacant lots | Plt | Fv | 26 | 128 | US | 0.0111 | 0.7371 | ha | Crowe 1979 | 4 | dd |
| invFFfPTt_Daily | Butterflies in forest frags | Inv | Ff | 8 | 38 | Costa Rica | 3 | 30 | ha | Daily & Erhlich 1995 | 2 | dd |
| brdFFfPTt_Daily | Birds in forest fragments | Brd | Ff | 8 | 131 | Costa Rica | 0.3 | 25 | ha | Daily et al 2001 | 3 | dd |
| mamWArPTw_Dalecky | Large mammals on FW reservoir island fragments | Ver | Af | 37 | 28 | French Guiana | 0.1 | 67 | ha | Dalecky et al 2002 | 2 | dd |
| Dami_2012 | birds on forest fragments | Brd | Ff | 31 | 134 | Nigeria | 0.3 | 40 | ha | Dami et al 2012 | 3 | fs |
| Dauber_2006 | Ants of grassland fragments | Inv | Fv | 22 | 24 | Sweden | 0.1 | 13.3 | ha | Dauber et al 2006 | 3 | fs |
| mamWFfPTt_deCastro | Small mammals in Altantic forest fragments | Ver | Ff | 8 | 11 | Brazil | 1.2 | 13.3 | ha | de Castro & Fernandez 2004 | 2 | dd |
| mamWFvUTt_Dickman | Mammals in urban parks | Ver | Fv | 50 | 20 | UK | 0.16 | 20 | ha | Dickman 1987 | 3 | dd |
| mamWFfPTt_Dinesen | Primates in forest fragments | Ver | Ff | 18 | 9 | Tanzania | 1 | 522 | km2 | Dinesen et al 2001 | 2 | dd |
| Ding_2013 | Birds of land-bridge island forest fragments | Brd | Af | 41 | 78 | China | 0.3 | 143.19 | ha | Ding et al 2013 | 3 | fs |
| pltSFfPTt_dosSantos | Trees in forest fragments | Plt | Ff | 10 | 239 | Brazil | 12.4 | 63.4 | ha | dos Santos et al 2007 | 4 | dd |
| invFFfPTt_dosSantos | Butterflies in forest reserves | Inv | Ff | 37 | 243 | Brazil | 7.4 | 84130 | ha | dos Santos et al 2018 | 1 | dd |
| Edwards_2010 | Plants on forest fragments | Plt | Ff | 12 | 79 | Malaysia | 0.7 | 87.1 | ha | Edwards et al 2010 | 4 | fs |
| invFFvPTt_Essl | Orthopterans in grassland fragments | Inv | Fv | 60 | 33 | Austria | 0.032 | 7 | ha | Essl & Dirnbock 2012 | 3 | dd |
| brdFArWTw_Feeley | Birds of reservoir island fragments | Brd | Af | 26 | 43 | Venezeula | 0.2 | 180 | ha | Feeley 2003 | 2 | dd |
| Fernandez_2013_a | Small mammals on shrubland/steppe fragments | Ver | Fv | 6 | 7 | Chile | 1.24 | 53.95 | ha | Fernandez & Simonetti 2013 | 4 | fs |
| brdFFvUTt_Fernandez | Birds in urban parks | Brd | Fv | 25 | 32 | Spain | 1 | 118.2 | ha | Fernandez-Juricic 2000 | 3 | dd |
| invFFfPTt_Filguerias | Dung beetles in forest frags | Inv | Ff | 18 | 21 | Brazil | 10 | 348 | ha | Filgueiras et al 2011 | 4 | dd |
| brdFFfPTt_Flaspoler | Birds in forest frags | Brd | Ff | 18 | 10 | Hawaii | 0.07 | 56 | ha | Flaspohler et al 2010 | 3 | dd |
| brdFFfPTt_Ford | Birds on woodlot fragments | Brd | Ff | 20 | 37 | UK - Oxford | 0.14 | 18 | ha | Ford 1987 | 2 | dd |
| Fujita_2008 | Ground beetles of forest fragments in urban matrix | Inv | Ff | 8 | 25 | Japan | 0.06 | 1.02 | ha | Fujita et al 2008 | 3 | fs |
| invWFfPTt_Galle | Spiders in forest frags, grassland matrix | Inv | Ff | 15 | 49 | Hungary | 0.01 | 5 | ha | Galle 2008 | 3 | dd |
| brdFFfPTt_Galli | Birds in forest fragments | Brd | Ff | 10 | 35 | US New Jersey | 0.01 | 24 | ha | Galli et al 1976 | 4 | dd |
| Garmendia_2013 | Mammals of forest fragments | Ver | Ff | 24 | 22 | Mexico | 2.4 | 75.9 | ha | Garmendia et al 2013 | 4 | fs |
| invWFfUTt_Gaublomme | Beetles in urban forest frags | Inv | Ff | 10 | 93 | Belgium | 5.3 | 88.2 | ha | Gaublomme et al 2008 | 3 | dd |
| Gavish_2012_a | Spiders of shrubland fragments | Inv | Fv | 12 | 114 | Israel | 0.11 | 3.9 | ha | Gavish et al 2012 | 3 | fs |
| Gavish_2012_b | Spiders of shrubland fragments | Inv | Fv | 12 | 116 | Israel | 0.06 | 2.81 | ha | Gavish et al 2012 | 3 | fs |
| Gavish_2012_c | Spiders of shrubland fragments | Inv | Fv | 12 | 100 | Israel | 0.16 | 4.24 | ha | Gavish et al 2012 | 3 | fs |
| Gibson_2013 | Small mammals on reservoir island fragments | Ver | Af | 16 | 12 | Thailand | 0.3 | 56.3 | ha | Gibson et al 2013 | 2 | fs |
| Giladi_2011_a | Plants of shrubland fragments | Plt | Fv | 11 | 87 | Israel | 0.18 | 3.9 | ha | Giladi et al 2011 | 3 | fs |
| Giladi_2011_b | Plants of shrubland fragments | Plt | Fv | 13 | 133 | Israel | 0.1 | 4.95 | ha | Giladi et al 2011 | 3 | fs |
| Giladi_2011_c | Plants of shrubland fragments | Plt | Fv | 14 | 176 | Israel | 0.16 | 4.24 | ha | Giladi et al 2011 | 3 | fs |
| Guadagnin_2005 | Birds on wetland fragments | Brd | Fv | 42 | 56 | Brazil | 0.19 | 145.24 | ha | Guadagnin et al 2005 | 3 | fs |
| brdFFfPTt_Haila1987 | Breeding birds, fragmented coniferous taiga | Brd | Ff | 34 | 45 | Finland | 0.4 | 101 | ha | Haila et al 1987 | 2 | dd |
| brdFFfPTt_Haila1993 | Breeding birds of forest fragments | Brd | Ff | 13 | 29 | Finland | 0.7 | 4.4 | ha | Haila et al 1993 | 2 | dd |
| pltSFfPTt_Hattori | Plants in temple conserved fragments | Plt | Ff | 38 | 177 | Japan | 0.003 | 7.84 | ha | Hattori & Ishida 2000 | 4 | dd |
| Henry_2007 | Bats of forest fragments | Ver | Af | 14 | 25 | French Guiana | 0.8 | 7.5 | ha | Henry et al 2007 | 4 | fs |
| brdFFfPTt_Holbech | Birds in logged forest fragments | Brd | Ff | 15 | 147 | Ghana | 23.6 | 587.6 | km2 | Holbech et al 2005 | 2 | dd |
| Horvath_2009 | Spiders of grassland fragments | Inv | Fv | 8 | 91 | Hungary | 2.3 | 353.5 | ha | Horvath et al 2009 | 4 | fs |
| brdFFfPTt_Howe | Birds in woodlot fragments | Brd | Ff | 9 | 26 | US, Wisconsin | 0.2 | 4.4 | ha | Howe & Jones 1977 | 3 | dd |
| pltSArWTw_Hu | Plants in reservoir island fragments | Plt | Af | 152 | 383 | China | 0.02 | 130 | ha | Hu et al 2012 | 3 | dd |
| pltSFfPTt_Ishida | Plants in temple conserved fragments | Plt | Ff | 29 | 119 | Japan | 0.025 | 16.4 | ha | Ishida et al 1998 | 4 | dd |
| Jauker_2019 | Bees and hoverflies of forest fragments | Inv | Ff | 32 | 132 | Germany | 0.03 | 5.13 | ha | Jauker et al 2019 | 4 | fs |
| Jung_2014 | Ground beetles of forest fragments | Inv | Ff | 10 | 30 | South Korea | 2.2 | 338.3 | ha | Jung et al 2014 | 4 | fs |
| Jyothi_2015 | Birds of sacred grove forest fragments | Brd | Ff | 15 | 108 | India | 1.6 | 24.4 | ha | Jyothi & Nameer 2015 | 3 | fs |
| Kapoor_2008 | Spiders of forest fragments | Inv | Ff | 9 | 135 | India | 11 | 200 | ha | Kapoor 2008 | 3 | fs |
| Kappes_2009 | Snails & slugs of forest fragments | Inv | Ff | 18 | 45 | Germany | 8 | 6000 | ha | Kappes et al 2009 | 4 | fs |
| mamWFfPTt_Kelt | Small mammals in fragmented forest | Ver | Ff | 14 | 9 | Chile | 1.5 | 125 | ha | Kelt 2000 | 3 | dd |
| hrpWFvPTt_Kitchener | Lizards in wheat belt reserves | Ver | Fv | 23 | 69 | Western Australia | 34 | 5119 | ha | Kitchener et al 1980a | 4 | dd |
| mamWFvMTt_Kitchener | Mammals in wheat belt reserves | Ver | Fv | 23 | 24 | Western Australia | 34 | 5119 | ha | Kitchener et al 1980b | 4 | dd |
| Knapp_2015_a | Beetles & spiders of forest fragments | Inv | Ff | 13 | 67 | Czech Republik | 0.01 | 0.89 | ha | Knapp & Řezáč 2015 | 4 | fs |
| Knapp_2015_b | Beetles & spiders of forest fragments | Inv | Ff | 13 | 82 | Czech Republik | 0.01 | 0.89 | ha | Knapp & Řezáč 2015 | 4 | fs |
| Lambert_2003 | Rodents on reservoir island fragments | Ver | Af | 9 | 6 | Venezuela | 0.23 | 11.1 | ha | Lambert_2003 | 4 | fs |
| Lima_2015 | amphibians of forest fragments | Ver | Af | 8 | 20 | Brazil | 3 | 207 | ha | Lima et al 2015 | 3 | fs |
| hrpWFfNTt_Lion | Reptiles in Atlantic Forest fragments | Ver | Ff | 23 | 15 | Brazil | 1.67 | 23.38 | ha | Lion et al 2016 | 4 | dd |
| Lion_2014 | Amphibians on forest fragments | Ver | Ff | 23 | 15 | Brazil | 1.71 | 27.41 | ha | Lion et al 2014 | 3 | fs |
| pltSAfWTw_Liu | Plants of reservoir island fragments | Plt | Af | 29 | 74 | China | 0.08 | 1154 | ha | Liu et al 2019 | 3 | dd |
| mamWFfPTt_Lomolino01 | Mammals in forest fragments | Ver | Ff | 20 | 18 | US | 0.93 | 58.9 | ha | Lomolino & Perault 2001 | 1 | dd |
| pltSFvPTt_Lumaret | Plants in isolated fragments | Plt | Fv | 8 | 193 | France | 0.0051 | 0.2172 | ha | Lumerat et al 1997 | 2 | dd |
| Manu_2007 | birds on forest fragments | Brd | Ff | 44 | 157 | Nigeria | 16 | 445 | ha | Manu et al 2007 | 3 | fs |
| brdFFfPTt_Marini | Birds in fragments | Brd | Ff | 6 | 145 | Brazil | 7.5 | 230 | ha | Marini 2001 | 3 | dd |
| mamWFfPTt_marshall | Monkeys in forest frags | Ver | Ff | 21 | 6 | Africa | 0.06 | 526 | km2 | Marshall et al 2010 | 2 | dd |
| Martensen_2012 | Birds on forest fragments | Brd | Ff | 53 | 119 | Brazil | 2.06 | 158.45 | ha | Martensen et al 2012 | 4 | fs |
| mamWFfPTt_Matthiae | Mammals in woodland fragments | Ver | Ff | 22 | 13 | US-Wisconsin | 0.4 | 40 | ha | Matthiae & Stearns 1981 | 2 | dd |
| brdFFfPTt_McCollin | Breeding birds fragmented woodlands | Brd | Ff | 16 | 49 | UK | 0.74 | 14.51 | ha | McCollin 1993 | 2 | dd |
| Meyer_2008 | Bats in forest fragments | Ver | Af | 11 | 30 | Panama | 2.5 | 50 | ha | Meyer & Kalko 2008 | 4 | fs |
| brdFFfPTt_Meynard | Birds on fragments | Brd | Ff | 9 | 51 | Chile | 180 | 60830 | ha | Meynard & Quinn 2008 | 4 | dd |
| invWFfUTt_Miyashita | Spiders in urban forest frags | Inv | Ff | 16 | 56 | Japan | 0.2 | 27 | ha | Miyashita et al 1998 | 3 | dd |
| brdFFfUTt_MohdAzlan | Birds of mangrove forest fragments, urban matrix | Brd | Ff | 13 | 70 | Australia | 0.67 | 594 | ha | Mohd-Azlan & Lawes 2011 | 3 | dd |
| Montgomery_2014 | Invertebrates on forest fragments | Inv | Ff | 15 | 58 | Malaysia | 5 | 720 | ha | Montgomery 2014 | 3 | fs |
| Nemesio_2007 | Euglossine bees of forest fragments | Inv | Ff | 4 | 15 | Brazil | 24.2 | 237 | ha | Nemesio_2007 | 4 | fs |
| Nemesio_2010 | Euglossine bees on forest fragments | Inv | Ff | 9 | 14 | Brazil | 1 | 353.8 | ha | Nemesio & Silveira 2010 | 4 | fs |
| brdFFfPTt_Newmark | Birds of tropical forest fragments | Brd | Ff | 9 | 23 | Tanzania | 0.1 | 30 | ha | Newmark 1991 | 3 | dd |
| Nogueira_2016 | Spiders of Atlantic forest fragments | Inv | Ff | 8 | 92 | South America | 14.1 | 175.1 | ha | Nogueira & Pinto-da-Rocha 2016 | 4 | fs |
| invFFvUTt_Nufio | Grasshoppers in grassland frags | Inv | Fv | 13 | 38 | US | 0.85 | 37.3 | ha | Nufio et al 2011 | 3 | dd |
| invFFfPTt_Nyeko | Dung beetles in forest frags | Inv | Ff | 6 | 45 | Uganda | 10 | 150 | ha | Nyeko 2009 | 3 | dd |
| Owen_2008 | Invertebrates on forest fragments | Inv | Ff | 33 | 42 | Great Britain | 0.38 | 36.25 | ha | Owen 2008 | 3 | fs |
| invFFfPTt_Pavuk | Beetles in woodland fragments | Inv | Ff | 7 | 26 | US-Ohio | 4.8 | 1241 | ha | Pavuk & Wadsworth 2012 | 4 | dd |
| hrpWFfPTt_Pineda | Anurans in cloud forest frags | Ver | Ff | 10 | 21 | Mexico | 11 | 122 | ha | Pineder & Halffter 2004 | 3 | dd |
| Raheem_2009 | Land snails of forest fragments | Inv | Ff | 20 | 55 | Sri Lanka | 1.17 | 10414.73 | ha | Raheem et al 2009 | 3 | fs |
| hrpWFfPTt_Ramana | Amphibians and reptiles of forest fragments | Ver | Ff | 7 | 59 | Madagascar | 25 | 63100 | ha | Ramanamanjato et al 2002 | 3 | dd |
| invWFfPTt_Ribas | Ants and crickets in forest remnants | Inv | Ff | 18 | 136 | Brazil | 3 | 299 | ha | Ribas et al 2005 | 3 | dd |
| mamWFfPTt_Rosenblatt | Mammals in forest fragments, row crop matrix | Ver | Ff | 10 | 16 | US | 1.8 | 600 | ha | Rosenblatt et al 1999 | 3 | dd |
| Savilaakso_2009 | Trees of forest fragments | Plt | Ff | 5 | 63 | Uganda | 0.93 | 16.9 | ha | Savilaakso et al 2009 | 3 | fs |
| hrpWFfPTt_Schlaepfer | Lizards & frogs in forest fragments | Ver | Ff | 7 | 16 | Costa Rica | 1.18 | 24.4 | ha | Schlaepfer & Gavin 2001 | 3 | dd |
| Schnitzler_2008_a | Hymenopteran parasitoids of forest fragments | Inv | Ff | 9 | 101 | New Zealand | 0.12 | 2.76 | ha | Schnitzler 2008 | 4 | fs |
| Schnitzler_2008_b | Plants of forest fragments | Plt | Ff | 10 | 117 | New Zealand | 0.12 | 2.76 | ha | Schnitzler 2008 | 4 | fs |
| invFFfPTt_Shreeve | Butterflies of isolated woodlands | Inv | Ff | 22 | 26 | UK | 2 | 175 | ha | Shreeve & Mason 1980 | 3 | dd |
| pltSFfPTt_Silva | Bryophytes in Atlantic forest fragments | Plt | Ff | 10 | 99 | Brazil | 23 | 2628 | ha | Silva & Porto 2009 | 4 | dd |
| mamWFfPTt_Silva | Small mammals in forest frags | Ver | Ff | 14 | 11 | Canada | 0.07 | 5.82 | ha | Silva 2001 | 3 | dd |
| Silveira_2015 | Euglossine beers of forest fragments | Inv | Ff | 5 | 14 | Brazil | 20 | 70 | ha | Silveira et al 2015 | 4 | fs |
| Slade_2013 | Macromoths of forest fragments | Inv | Ff | 9 | 85 | Great Britain | 0.37 | 21.72 | ha | Slade et al 2013 | 4 | fs |
| hrpWFvPTt_Smith | Lizards of semi-arid frags | Ver | Fv | 26 | 15 | Western Australia | 0.5 | 138 | ha | Smith et al 1996 | 4 | dd |
| brdFFvUTt_Soule | Chapparal birds, urban frags | Brd | Fv | 31 | 8 | US San Diego | 0.4 | 102.8 | ha | Soule et al 1988 | 2 | dd |
| Sridhar_2008 | Mammals of forest fragments | Ver | Ff | 9 | 15 | India | 32 | 2600 | ha | Sridhar et al 2008 | 3 | fs |
| Stireman_2014 | Caterpillars of forest fragments | Inv | Ff | 19 | 158 | USA | 6.2 | 802.3 | ha | Stireman et al 2014 | 4 | fs |
| Struebig_2008 | Bats of forest fragments | Ver | Ff | 15 | 36 | Malaysia | 31 | 11339 | ha | Struebig et al 2008 | 3 | fs |
| invWFvPTt_Suarez | Ants in veg. fragments | Inv | Fv | 40 | 45 | US | 0.4 | 101.6 | ha | Suarez et al 1998 | 3 | dd |
| Ulrich_2016 | birds on forest fragments | Brd | Ff | 12 | 70 | Kenya | 1.14 | 179 | ha | Ulrich et al 2016 | 2 | fs |
| invFFfPTt_Usher | Moths, artificial woodland fragments | Inv | Ff | 18 | 214 | UK | 0.5 | 30.6 | ha | Usher & Keiller 1998 | 3 | dd |
| hrpWFfNTt_Vallan | Amphibians in fragmented forest | Ver | Ff | 6 | 24 | Madagascar | 0.16 | 136 | ha | Vallan 2000 | 3 | dd |
| Vasconcelos_2006 | Mammals on forest fragments | Ver | Ff | 24 | 115 | Brazil | 2.39 | 361 | ha | Vasconcelos et al 2006 | 4 | fs |
| Vulinec_2008 | Invertebrates on forest fragments | Inv | Ff | 4 | 15 | Brazil | 8.5 | 66.4 | ha | Vulinec et al 2008 | 4 | fs |
| brdFArWTw_Wang | Birds of reservoir island fragments | Brd | Af | 42 | 93 | China | 0.3 | 1289.2 | ha | Wang et al 2010 | 3 | dd |
| brdFFfPTt_Wang | Small mammals of forest fragments | Brd | Ff | 20 | 60 | China | 0.97 | 1289.2 | ha | Wang et al 2010 | 3 | dd |
| mamWArWTw_Wang | Mammals of reservoir island fragments | Ver | Af | 14 | 11 | China | 0.97 | 1289.2 | ha | Wang et al 2010 | 3 | dd |
| brdFFfPTt_Watson | Birds of forest fragments | Brd | Ff | 16 | 60 | Mexico | 6 | 159246 | ha | Watson 2003 | 3 | dd |
| pltSFfPTt_Weaver | Plants of forest frags | Plt | Ff | 10 | 30 | Canada | 0.82 | 8.11 | ha | Weaver & Kellman 1981 | 3 | dd |
| invWFfUTt_Weller | Beetles of urban forest frags | Inv | Ff | 9 | 23 | Germany | 3 | 160 | ha | Weller & Ganzhorn 2003 | 4 | dd |
| Williams_2011 | Invertebrates of forest fragments | Inv | Ff | 46 | 34 | Australia | 0.7 | 362 | ha | Williams 2011 | 3 | fs |
| brdFFfPTt_Willson | Birds of forest frags | Brd | Ff | 11 | 25 | Chile | 0.49 | 350 | ha | Wilson et al 1994 | 3 | dd |
| brdFArWTw_Wright | Birds of reservoir island fragments | Brd | Af | 6 | 50 | Panama | 7.6 | 76.5 | ha | Wright 1985 | 3 | dd |

# Data source publications

Aguiar, W. M. D., and M. C. Gaglianone, (2012). Euglossine bee communities in small forest fragments of the Atlantic Forest, Rio de Janeiro state, southeastern Brazil (Hymenoptera, Apidae). Revista Brasileira de Entomologia 56:210–219.

Aizen, M. A., and P. Feinsinger. (1994). Habitat Fragmentation, Native Insect Pollinators, and Feral Honey Bees in Argentine “Chaco Serrano.” Ecological Applications 4:378–392.

Almeida-Gomes, M., and C. F. D. Rocha, (2014). Diversity and distribution of lizards in fragmented Atlantic Forest landscape in Southeastern Brazil. Journal of Herpetology 48:423–429.

Anciaes, M. & Marini, M.A. (2000) The effects of fragmentation on fluctuating asymmetry in passerine birds of Brazilian tropical forests. Journal of Applied Ecology, 37, 1013-1028.

Báldi, A. & Kisbenedek, T. (1997) Orthopteran assemblages as indicators of grassland naturalness in Hungary. Agriculture Ecosystems & Environment, 66, 121-129.

Bell, K. E., and M. A. Donnelly. (2006). Influence of forest fragmentation on community structure of frogs and lizards in northeastern Costa Rica. Conservation Biology 20:1750–1760.

Benedick, S., Hill, J.K., Mustaffa, N., Chey, V.K., Maryati, M., Searle, J.B., Schilthuizen, M. & Hamer, K.C. (2006) Impacts of rain forest fragmentation on butterflies in northern Borneo: species richness, turnover and the value of small fragments. Journal of Applied Ecology, 43, 967-977.

Berg, Å. (1997). Diversity and abundance of birds in relation to forest fragmentation, habitat quality and heterogeneity. Bird Study 44:355–366.

Bernard, E., and M. B. Fenton. (2007). Bats in a fragmented landscape: Species composition, diversity and habitat interactions in savannas of Santarem, Central Amazonia, Brazil. Biological Conservation 134:332–343.

Blake, J.G. (1991) Nested subsets and the distribution of birds on isolated woodlots. Conservation Biology, 5, 58-66.

Bolger, D.T., Alberts, A.C., Sauvajot, R.M., Potenza, P., McCalvin, C., Tran, D., Mazzoni, S. & Soule, M.E. (1997) Response of rodents to habitat fragmentation in coastal southern California. Ecological Applications, 7, 552-563.

Bond, R.R. (1957) Ecological distribution of breeding birds in the upland forests of southern Wisconsin. Ecological Monographs, 27, 352-384.

Bragagnolo, C., A. A. Nogueira, R. Pinto-da-Rocha, and R. Pardini. (2007). Harvestmen in an Atlantic forest fragmented landscape: Evaluating assemblage response to habitat quality and quantity. Biological Conservation 139:389–400.

Brosi, B. J., G. C. Daily, T. M. Shih, F. Oviedo, and G. Durán. (2008). The effects of forest fragmentation on bee communities in tropical countryside. Journal of Applied Ecology 45:773–783.

Brosi, B. J., (2009). The effects of forest fragmentation on euglossine bee communities (Hymenoptera: Apidae: Euglossini). Biological Conservation 142:414–423.

Cabrera-Guzman, E. & Reynoso, V.H. (2012) Amphibian and reptile communities of rainforest fragments: minimum patch size to support high richness and abundance. Biodiversity and Conservation, 21, 3243-3265.

Capizzi, D., Luiselli, L. & Papi, R. (2015) Temporal changes in Mediterranean bird communities across fragmented and continuous forests. Ecological Research, 30, 615-624.

Castelletta, M., Thiollay, J.M. & Sodhi, N.S. (2005) The effects of extreme forest fragmentation on the bird community of Singapore Island. Biological Conservation, 121, 135-155.

Cayuela, L., D. J. Golicher, J. M. R. Benayas, M. González-Espinosa, and N. Ramírez-Marcial. (2006). Fragmentation, disturbance and tree diversity conservation in tropical montane forests. Journal of Applied Ecology 43:1172–1181.

Charles, J.K. & Ang, B.B. (2010) Non-volant small mammal community responses to fragmentation of kerangas forests in Brunei Darussalam. Biodiversity and Conservation, 19, 543-561.

Crooks, K.R. (2002) Relative sensitivities of mammalian carnivores to habitat fragmentation. Conservation Biology, 16, 488-502.

Crooks, K.R., Suarez, A.V., Bolger, D.T. & Soule, M.E. (2001) Extinction and colonization of birds on habitat islands. Conservation Biology, 15, 159-172.

Crowe, T.M. (1979) Lots of weeds - insular phytogeography of vacant urban lots. Journal of Biogeography, 6, 169-181.

Daily, G.C. & Ehrlich, P.R. (1995) Preservation of biodiversity in small rain-forest patches - rapid evaluations using butterfly trapping. Biodiversity and Conservation, 4, 35-55.

Daily, G.C., Ehrlich, P.R. & Sanchez-Azofeifa, G.A. (2001) Countryside biogeography: Use of human-dominated habitats by the avifauna of southern Costa Rica. Ecological Applications, 11, 1-13.

Dalecky, A., Chauvet, S., Ringuet, S., Claessens, O., Judas, J., Larue, M. & Cosson, J.F. (2002) Large mammals on small islands: Short term effects of forest fragmentation on the large mammal fauna in French Guiana. Revue D Ecologie-La Terre Et La Vie, 145-164.

Dami, F. D., G. S. Mwansat, and S. A. Manu. (2013). The effects of forest fragmentation on species richness on the Obudu Plateau, south-eastern Nigeria. African Journal of Ecology 51:32–36.

Dauber, J., J. Bengtsson, and L. Lenoir. (2006). Evaluating effects of habitat loss and landuse continuity on ant species richness in seminatural grassland remnants. Conservation Biology 20:1150–1160.

de Castro, E.B.V. & Fernandez, F.A.S. (2004) Determinants of differential extinction vulnerabilities of small mammals in Atlantic forest fragments in Brazil. Biological Conservation, 119, 73-80.

Dickman, C.R. (1987) Habitat fragmentation and vertebrate species richness in an urban environment. Journal of Applied Ecology, 24, 337-351.

Dinesen, L., Lehmberg, T., Rahner, M.C. & Fjeldsa, J. (2001) Conservation priorities for the forests of the Udzungwa Mountains, Tanzania, based on primates, duikers and birds. Biological Conservation, 99, 223-236.

Ding, C.Z., Jiang, X.M., Xie, Z.C. & Brosse, S. (2017) Seventy-five years of biodiversity decline of fish assemblages in Chinese isolated plateau lakes: widespread introductions and extirpations of narrow endemics lead to regional loss of dissimilarity. Diversity and Distributions, 23, 171-184.

dos Santos, J.P., Freitas, A.V.L., Brown, K.S., Carreira, J.Y.O., Gueratto, P.E., Rosa, A.H.B., Lourenco, G.M., Accacio, G.M., Uehara-Prado, M., Iserhard, C.A., Richter, A., Gawlinski, K., Romanowski, H.P., Mega, N.O., Teixeira, M.O., Moser, A., Ribeiro, D.B., Araujo, P.F., Filgueiras, B.K.C., Melo, D.H.A., Leal, I.R., Beirao, M.D., Ribeiro, S.P., Cambui, E.C.B., Vasconcelos, R.N., Cardoso, M.Z., Paluch, M., Greve, R.R., Voltolini, J.C., Galetti, M., Regolin, A.L., Sobral-Souza, T. & Ribeiro, M.C. (2018) Atlantic butterflies: a data set of fruit-feeding butterfly communities from the Atlantic forests. Ecology, 99, 2875-2875.

dos Santos, K., Kinoshita, L.S. & dos Santos, F.A.M. (2007) Tree species composition and similarity in semideciduous forest fragments of southeastern Brazil. Biological Conservation, 135, 268-277.

Edwards, D. P., J. A. Hodgson, K. C. Hamer, S. L. Mitchell, A. H. Ahmad, S. J. Cornell, and D. S. Wilcove. (2010). Wildlife-friendly oil palm plantations fail to protect biodiversity effectively. Conservation Letters 3:236–242.

Essl, F. & Dirnbock, T. (2012) What determines Orthoptera species distribution and richness in temperate semi-natural dry grassland remnants? Biodiversity and Conservation, 21, 2525-2537.

Feeley, K. (2003) Analysis of avian communities in Lake Guri, Venezuela, using multiple assembly rule models. Oecologia, 137, 104-113.

Fernandez-Juricic, E. (2000) Bird community composition patterns in urban parks of Madrid: The role of age, size and isolation. Ecological Research, 15, 373-383.

Filgueiras, B.K.C., Iannuzzi, L. & Leal, I.R. (2011) Habitat fragmentation alters the structure of dung beetle communities in the Atlantic Forest. Biological Conservation, 144, 362-369.

Flaspohler, D.J., Giardina, C.P., Asner, G.P., Hart, P., Price, J., Lyons, C.K. & Castaneda, X. (2010) Long-term effects of fragmentation and fragment properties on bird species richness in Hawaiian forests. Biological Conservation, 143, 280-288.

Ford, H.A. (1987) Bird communities on habitat islands in England. Bird Study, 34, 205-218.

Fujita, A., K. Maeto, Y. Kagawa, and N. Ito. (2008). Effects of forest fragmentation on species richness and composition of ground beetles (Coleoptera: Carabidae and Brachinidae) in urban landscapes. Entomological Science 11:39–48.

Galle, R. (2008) The effect of a naturally fragmented landscape on the spider assemblages. North-Western Journal of Zoology, 4, 61-71.

Galli, A.E., Leck, C.F. & Forman, R.T.T. (1976) Avian distribution patterns in forest islands of different sizes in central New Jersey. Auk, 93, 356-364.

Garmendia, A., V. Arroyo‐Rodriguez, A. Estrada, E. J. Naranjo & K. E. Stoner. (2013). Landscape and patch attributes impacting medium‐ and large‐sized terrestrial mammals in a fragmented rain forest. Journal of Tropical Ecology, 29:331– 344.

Gaublomme, E., Hendrickx, F., Dhuyvetter, H. & Desender, K. (2008) The effects of forest patch size and matrix type on changes in carabid beetle assemblages in an urbanized landscape. Biological Conservation, 141, 2585-2596.

Gavish, Y., Ziv, Y. & Rosenzweig, M.L. (2012) Decoupling Fragmentation from Habitat Loss for Spiders in Patchy Agricultural Landscapes. Conservation Biology, 26, 150-159.

Gibson, L., A. J. Lynam, C. J. A. Bradshaw, F. He, D. P. Bickford, D. S. Woodruff, S. Bumrungsri, and W. F. Laurance. (2013). Near-Complete Extinction of Native Small Mammal Fauna 25 Years After Forest Fragmentation. Science 341:1508–1510.

Giladi, I., Y. Ziv, F. May, and F. Jeltsch. (2011). Scale-dependent determinants of plant species richness in a semi-arid fragmented agro-ecosystem. Journal of Vegetation Science 22:983–996.

Guadagnin, D. L., Â. S. Peter, L. F. C. Perello, and L. Maltchik. (2005). Spatial and Temporal Patterns of Waterbird Assemblages in Fragmented Wetlands of Southern Brazil. Waterbirds 28:261–272.

Haila, Y., Hanski, I.K. & Raivio, S. (1987) Breeding bird distribution in fragmented coniferous taiga in southern Finland. Ornis Fennica, 64, 90-106.

Haila, Y., Hanski, I.K. & Raivio, S. (1993) Turnover of breeding birds in small forest fragments - the sampling colonization hypothesis corroborated. Ecology, 74, 714-725.

Hattori, T. & Ishida, H. (2000) Relationship between species diversity, species composition and forest area of fragmented lucidophyllous forests in central Miyazaki Prefecture. Japanese Journal of Ecology (Otsu), 50, 221-234.

Henry, M., J. M. Pons, and J. F. Cosson. (2007). Foraging behaviour of a frugivorous bat helps bridge landscape connectivity and ecological processes in a fragmented rainforest. Journal of Animal Ecology 76:801–813.

Holbech, L.H. (2005) The implications of selective logging and forest fragmentation for the conservation of avian diversity in evergreen forests of south-west Ghana. Bird Conservation International, 15, 27-52.

Horváth, R., T. Magura, C. Szinetár, and B. Tóthmérész. (2009). Spiders are not less diverse in small and isolated grasslands, but less diverse in overgrazed grasslands: A field study (East Hungary, Nyírség). Agriculture, Ecosystems and Environment 130:16–22.

Howe, R.W. & Jones, G. (1977) Avian utilization of small woodlots in Dane County, Wisconsin. Passenger Pigeon, 39, 313-319,illust.

Hu, G., Wu, J.G., Feeley, K.J., Xu, G.F. & Yu, M.J. (2012) The Effects of Landscape Variables on the Species-Area Relationship during Late-Stage Habitat Fragmentation. Plos One, 7

Ishida, H., Hattori, T., Takeda, Y. & Kodate, S. (1998) Relationship between species richness or species composition and area of fragmented lucidophyllous forests in southeastern Hyogo Prefecture. Japanese Journal of Ecology (Tokyo), 48, 1-16.

Jauker, F., B. Jauker, I. Grass, I. S. Dewenter, and V. Wolters. (2019). Partitioning wild bee and hoverfly contributions to plant-pollinator network structure in fragmented habitats Ecology 100:e02569.

Jung, J. K., S. T. Kim, S. Y. Lee, C. G. Park, J. K. Park, and J. H. Lee. (2014). A comparison of diversity and species composition of ground beetles (Coleoptera: Carabidae) between conifer plantations and regenerating forests in Korea. Ecological Research 29:877–887.

Jyothi, K. M., and P. O. Nameer. (2015). Birds of sacred groves of northern Kerala. Indian Journal of Threatened Taxa 7:8226–8236.

Kapoor, V. (2008). Effects of rainforest fragmentation and shade-coffee plantations on spider communities in the Western Ghats, Indian Journal of Insect Conservation 12:53–68.

Kappes, H., K. Jordaens, F. Hendrickx, J. P. Maelfait, L. Lens, and T. Backeljau. (2009). Response of snails and slugs to fragmentation of lowland forests in NW Germany. Landscape Ecology 24:685–697.

Kelt, D.A. (2000) Small mammal communities in rainforest fragments in Central Southern Chile. Biological Conservation, 92, 345-358.

Kitchener, D.J., Chapman, A., Muir, B.G. & Palmer, M. (1980a) The conservation value for mammals of reserves in the western Australian wheatbelt. Biological Conservation, 18, 179-207.

Kitchener, D.J., Chapman, A., Dell, J. & Muir, B.G. (1980b) Lizard assemblage and reserve size and structure in the western Australian wheatbelt - some implications for conservation. Biological Conservation, 17, 25-62.

Knapp, M., and M. Řezáč. (2015). Even the smallest non-crop habitat islands could be beneficial: Distribution of carabid beetles and spiders in agricultural landscape. PLoS ONE 10:e0123052.

Lambert, T. D., G. H. Adler, C. M. Riveros, L. Lopez, R. Ascanio, and J. Terborgh, (2003). Rodents on tropical land-bridge islands. Journal of Zoology 260:179–187.

Lima, J. R., U. Galatti, C. J. Lima, S. B. Fáveri, H. L. Vasconcelos, and S. Neckel-Oliveira. (2015). Amphibians on Amazonian land-bridge islands are affected more by area than isolation. Biotropica 47:369–376.

Lion, M. B., A. A. Garda, and C. R. Fonseca. (2014). Split distance: A key landscape metric shaping amphibian populations and communities in forest fragments. Diversity and Distributions 20:1245–1257.

Lion, M. B., A. A. Garda, D. J. Santana, and C. R. Fonseca. (2016). The conservation value of small fragments for Atlantic forest reptiles. Biotropica 48:265–275.

Liu, J.L., Matthews, T.J., Zhong, L., Liu, J.J., Wu, D.H. & Yu, M.J. (2019) Environmental filtering underpins the island species-area relationship in a subtropical anthropogenic archipelago. Journal of Ecology

Lomolino, M.V. & Perault, D.R. (2001) Island biogeography and landscape ecology of mammals inhabiting fragmented, temperate rain forests. Global Ecology and Biogeography, 10, 113-132.

Lumaret, R., Guillerm, J.L., Maillet, J. & Verlaque, R. (1997) Plant species diversity and polyploidy in islands of natural vegetation isolated in extensive cultivated lands. Biodiversity and Conservation, 6, 591-613.

Manu, S., W. Peach, and W. Cresswell. (2007). The effects of edge, fragment size and degree of isolation on avian species richness in highly fragmented forest in West Africa. Ibis 149:287–297.

Marini, M.A. (2001) Effects of forest fragmentation on birds of the cerrado region, Brazil. Bird Conservation International, 11, 13-25.

Marshall, A.R., Jorgensbye, H.I.O., Rovero, F., Platts, P.J., White, P.C.L. & Lovett, J.C. (2010) The Species Area Relationship and Confounding Variables in a Threatened Monkey Community. American Journal of Primatology, 72, 325-336.

Martensen, A. C., M. C. Ribeiro, C. Banks-Leite, P. I. Prado, and J. P. Metzger. (2012). Associations of Forest Cover, Fragment Area, and Connectivity with Neotropical Understory Bird Species Richness and Abundance. Conservation Biology 26:1100–1111.

Matthiae, P.E. & Stearns, F. (1981) Mammals in forest islands in southeastern Wisconsin. Ecological Studies, 41, 55-66.

McCollin, D. (1993) Avian distribution patterns in a fragmented wooded landscape (North Humberside, UK) - the role of between-patch and within-patch structure. Global Ecology and Biogeography Letters, 3, 48-62.

Meyer, C. F. J., and E. K. V Kalko. (2008). Assemblage-level responses of phyllostomid bats to tropical forest fragmentation: Land-bridge islands as a model system. Journal of Biogeography 35:1711–1726.

Meynard, C.N. & Quinn, J.F. (2008) Bird metacommunities in temperate South American forest: Vegetation structure, area, and climate effects. Ecology, 89, 981-990.

Miyashita, T., Shinkai, A. & Chida, T. (1998) The effects of forest fragmentation on web spider communities in urban areas. Biological Conservation, 86, 357-364.

Mohd-Azlan, J. & Lawes, M.J. (2011) The effect of the surrounding landscape matrix on mangrove bird community assembly in north Australia. Biological Conservation, 144, 2134-2141.

Montgomery, M. J. (2014). Assessing biodiversity and ecosystem functioning in fragmented tropical landscapes. Doctoral Thesis at the University of York, Great Britain.

Nemésio, A., and F. A. Silveira. (2007). Orchid bee fauna (Hymenoptera: Apidae: Euglossina) of Atlantic Forest fragments inside an urban area in southeastern Brazil. Neotropical Entomology 36:186–191.

Nemésio, A., and F. A. Silveira. (2010). Forest fragments with larger core areas better sustain diverse orchid bee faunas (Hymenoptera: Apidae: Euglossina). Neotropical Entomology 39:555–561.

Newmark, W.D. (1991) Tropical forest fragmentation and the local extinction of understory birds in the eastern Usambara Mountains, Tanzania. Conservation Biology, 5, 67-78.

Nogueira, A., and R. Pinto-da-Rocha. (2016). The effects of habitat size and quality on the orb weaving spider guild (Arachnida: Araneae) in an Atlantic Forest fragmented landscape. Journal of Arachnology 44:36–45.

Nufio, C.R., McClenahan, J.L. & Bowers, M.D. (2011) Grasshopper response to reductions in habitat area as mediated by subfamily classification and life history traits. Journal of Insect Conservation, 15, 409-419.

Nyeko, P. (2009) Dung Beetle Assemblages and Seasonality in Primary Forest and Forest Fragments on Agricultural Landscapes in Budongo, Uganda. Biotropica, 41, 476-484.

Owen, C. L. (2008). Mapping biodiversity in a modified landscape. Master of Sciences Thesis at the Imperial College London, Great Britain.

Pavuk, D.M. & Wadsworth, A.M. (2012) Longhorned beetle (Coleoptera: Cerambycidae) diversity in a fragmented temperate forest landscape. F1000Research, 1, 25.

Pineda, E. & Halffter, G. (2004) Species diversity and habitat fragmentation: frogs in a tropical montane landscape in Mexico. Biological Conservation, 117, 499-508.

Raheem, D. C., F. Naggs, P. D. James Chimonides, R. C. Preece, and P. Eggleton. (2009). Fragmentation and pre-existing species turnover determine land-snail assemblages of tropical rain forest. Journal of Biogeography 1083 36:1923–1938.

Ramanamanjato, J.B., McIntyre, P.B. & Nussbaum, R.A. (2002) Reptile, amphibian, and lemur diversity of the Malahelo Forest, a biogeographical transition zone in southeastern Madagascar. Biodiversity and Conservation, 11, 1791-1807.

Ribas, C.R., Sobrinho, T.G., Schoereder, J.H., Sperber, C.F., Lopes-Andrade, C. & Soares, S.M. (2005) How large is large enough for insects? Forest fragmentation effects at three spatial scales. Acta Oecologica-International Journal of Ecology, 27, 31-41.

Rosenblatt, D.L., Heske, E.J., Nelson, S.L., Barber, D.H., Miller, M.A. & MacAllister, B. (1999) Forest fragments in east-central Illinois: Islands or habitat patches for mammals? American Midland Naturalist, 141, 115-123.

Savilaakso, S., J. Koivisto, T. O. Veteli, and H. Roininen. (2009). Microclimate and tree community linked to differences in lepidopteran larval communities between forest fragments and continuous forest. Diversity and Distributions 15:356–365.

Schlaepfer, M.A. & Gavin, T.A. (2001) Edge effects on lizards and frogs in tropical forest fragments. Conservation Biology, 15, 1079-1090.

Schnitzler, F. R. (2008). Hymenopteran parasitoid diversity and tri-trophic interactions: the effects of habitat fragmentation in Wellington, New Zealand. PhD thesis, Victoria University of Wellington, New Zealand.

Shreeve, T.G. & Mason, C.F. (1980) The number of butterfly species in woodlands. Oecologia, 45, 414-418.

Silva, M. (2001) Abundance, diversity, and community structure of small mammals in forest fragments in Prince Edward Island National Park, Canada. Canadian Journal of Zoology-Revue Canadienne De Zoologie, 79, 2063-2071.

Silva, M.P.P. & Porto, K.C. (2009) Effect of fragmentation on the community structure of epixylic bryophytes in Atlantic Forest remnants in the Northeast of Brazil. Biodiversity and Conservation, 18, 317-337.

Silveira, G. C., R. F. Freitas, T. H. A. Tosta, L. S. Rabelo, M. C. Gaglianone, and S. C. Augusto. (2015). The orchid bee fauna in the Brazilian savanna: do forest formations contribute to higher species diversity? Apidologie 46:197–208.

Slade, E. M., T. Merckx, T. Riutta, D. P. Bebber, D. Redhead, P. Riordan, D. W. Macdonald, and P. Bebber. (2013). Life-history traits and landscape characteristics predict macromoth responses to forest fragmentation. Ecology 94:1519–1530.

Smith, G.T., Arnold, G.W., Sarre, S., AbenspergTraun, M. & Steven, D.E. (1996) The effects of habitat fragmentation and livestock-grazing on animal communities in remnants of gimlet Eucalyptus salubris woodland in the Western Australian wheatbelt .2. Lizards. Journal of Applied Ecology, 33, 1302-1310.

Soule, M.E., Bolger, D.T., Alberts, A.C., Wright, J., Sorice, M. & Hill, S. (1988) Reconstructed dynamics of rapid extinctions of chaparral-requiring birds in urban habitat islands. Conservation Biology, 2, 75-92.

Sridhar, H., T. S. Raman, and D. Mudappa. (2008). Mammal persistence and abundance in tropical rainforest remnants in the southern Western Ghats, Indian Current Science 94:748–757.

Stireman, J. O., H. Devlin, and A. L. Doyle. (2014). Habitat fragmentation, tree diversity, and plant invasion interact to structure forest caterpillar communities. Oecologia 176:207–224.

Struebig, M. J., T. Kingston, A. Zubaid, A. Mohd-Adnan, and S. J. Rossiter. (2008). Conservation value of forest fragments to Palaeotropical bats. Biological Conservation 141:2112–2126.

Suarez, A.V., Bolger, D.T. & Case, T.J. (1998) Effects of fragmentation and invasion on native ant communities in coastal southern California. Ecology, 79, 2041-2056.

Ulrich, W., L. Lens, J. A. Tobias, and J. C. Habel. (2016). Contrasting patterns of species richness and functional diversity in bird communities of east African cloud forest fragments. PLoS ONE 11:e0163338.

Usher, M.B. & Keiller, S.W.J. (1998) The macrolepidoptera of farm woodlands: determinants of diversity and community structure. Biodiversity and Conservation, 7, 725-748.

Vallan, D. (2000) Influence of forest fragmentation on amphibian diversity in the nature reserve of Ambohitantely, highland Madagascar. Biological Conservation, 96, 31-43.

Vasconcelos, H. L., J. M. S. Vilhena, W. E. Magnusson, and A. L. K. M. Albernaz. (2006) Long-term effects of forest fragmentation on Amazonian ant communities. Journal of Biogeography 33:1348–1356.

Vulinec, K., A. P. Lima, E. A. R. Carvalho, and D. J. Mellow. (2008). Dung Beetles and Long term Habitat Fragmentation in Alter do Chao, Amazonia, Brazil. Tropical Conservation Science 1:111–121.

Wang, Y.P., Bao, Y.X., Yu, M.J., Xu, G.F. & Ding, P. (2010) Nestedness for different reasons: the distributions of birds, lizards and small mammals on islands of an inundated lake. Diversity and Distributions, 16, 862-873.

Watson, D.M. (2003) Long-term consequences of habitat fragmentation - highland birds in Oaxaca, Mexico. Biological Conservation, 111, 283-303.

Weaver, M. & Kellman, M. (1981) The effects of forest fragmentation on woodlot tree biotas in southern Ontario. Journal of Biogeography, 8, 199-210.

Weller, B. & Ganzhorn, J.U. (2004) Carabid beetle community composition, body size, and fluctuating asymmetry along an urban-rural gradient. Basic and Applied Ecology, 5, 193-201.

Williams, M. R. (2011). Habitat resources, remnant vegetation condition and area determine distribution patterns and abundance of butterflies and day-flying moths in a fragmented urban landscape, south-west Western Australia. Journal of Insect Conservation 15:37–54.

Wilson, M.F., De Santo, T.L., Sabag, C. & Armesto, J.J. (1994) Avian communities of fragmented south-temperate rainforests in Chile. Conservation Biology, 8, 508-520.

Wright, S.J. (1985) How isolation affects rates of turnover of species on islands. Oikos, 44, 331-340.
